# Transcriptome prediction performance across machine learning models and diverse ancestries

**DOI:** 10.1016/j.xhgg.2020.100019

**Published:** 2021-01-05

**Authors:** Paul C. Okoro, Ryan Schubert, Xiuqing Guo, W. Craig Johnson, Jerome I. Rotter, Ina Hoeschele, Yongmei Liu, Hae Kyung Im, Amy Luke, Lara R. Dugas, Heather E. Wheeler

**Affiliations:** 1 Program in Bioinformatics, Loyola University Chicago, Chicago, IL, USA;; 2 Department of Mathematics and Statistics, Loyola University Chicago, Chicago, IL, USA;; 3 Institute for Translational Genomics and Population Sciences, The Lundquist Institute and Department of Pediatrics at Harbor-UCLA Medical Center, Torrance, CA, USA;; 4 Department of Biostatistics, University of Washington, Seattle, WA, USA;; 5 Fralin Life Sciences Institute, Virginia Tech, Blacksburg, VA, USA;; 6 Department of Statistics, Virginia Tech, Blacksburg, VA, USA;; 7 Wake Forest School of Medicine, Winston-Salem, NC, USA;; 8 Department of Medicine, Duke University School of Medicine, Durham, NC, USA;; 9 Section of Genetic Medicine, Department of Medicine, University of Chicago, Chicago, IL, USA;; 10 Department of Public Health Sciences, Parkinson School of Health Sciences and Public Health, Loyola University Chicago, Maywood, IL, USA;; 11 Department of Human Biology, Faculty of Health Sciences, University of Cape Town, Cape Town, South Africa;; 12 Department of Biology, Loyola University Chicago, Chicago, IL, USA;; 13 Department of Computer Science, Loyola University Chicago, Chicago, IL, USA

## Abstract

Transcriptome prediction methods such as PrediXcan and FUSION have become popular in complex trait mapping. Most transcriptome prediction models have been trained in European populations using methods that make parametric linear assumptions like the elastic net (EN). To potentially further optimize imputation performance of gene expression across global populations, we built transcriptome prediction models using both linear and non-linear machine learning (ML) algorithms and evaluated their performance in comparison to EN. We trained models using genotype and blood monocyte transcriptome data from the Multi-Ethnic Study of Atherosclerosis (MESA) comprising individuals of African, Hispanic, and European ancestries and tested them using genotype and whole-blood transcriptome data from the Modeling the Epidemiology Transition Study (METS) comprising individuals of African ancestries. We show that the prediction performance is highest when the training and the testing population share similar ancestries regardless of the prediction algorithm used. While EN generally outperformed random forest (RF), support vector regression (SVR), and K nearest neighbor (KNN), we found that RF outperformed EN for some genes, particularly between disparate ancestries, suggesting potential robustness and reduced variability of RF imputation performance across global populations. When applied to a high-density lipoprotein (HDL) phenotype, we show including RF prediction models in PrediXcan revealed potential gene associations missed by EN models. Therefore, by integrating other ML modeling into PrediXcan and diversifying our training populations to include more global ancestries, we may uncover new genes associated with complex traits.

## Introduction

Advancements in high-throughput genotyping and sequencing technologies have led to an explosion in the amount of genetic data publicly available.^1^ Leveraging these technological successes, genome-wide association studies (GWASs) have continued to uncover thousands of genetic variants that are associated with different complex traits in humans.^2^ However, most of these variants identified through GWAS are usually found in the noncoding region of the genome, thereby complicating identification of their functional importance in understanding the biology of complex traits.^1–4^ Many studies have shown that these regions are particularly enriched for gene regulatory variants such as expression quantitative loci (eQTLs), and thus genetically regulated gene expression might play a critical role in explaining the phenotypic variability in a wide range of complex traits.^5–9^ More so, given that a handful of SNPs have large effect associations that can explain most of the heritable component of gene expression traits, mathematical modeling of the relationship between genotype and gene expression is achievable using moderate sample sizes.^10^ Indeed, this has led to the development of transcriptome methods such as PrediXcan^11^ and FUSION,^12^ which integrate *cis*-eQTL genotype and transcriptome datasets in order to predict the transcriptome from GWAS data and subsequently test for association between the predicted transcriptome and trait of interest. Unlike traditional GWASs, these gene-based approaches combine multiple SNPs into one functional unit and point directly to a biological mechanism, that is, either increased or decreased expression of a particular gene is associated with a trait. Because most GWASs lack corresponding transcriptome data, these methods may identify gene regulatory mechanisms underlying complex traits.

More specifically, the mathematical model used in PrediXcan is elastic net (EN),^13^ while FUSION uses Bayesian sparse linear mixed model (BSLMM).^14^ The EN model used by PrediXcan is a combination of L1 (LASSO)^15^ and L2 (Ridge)^16^ regularization of the cis-eQTL effect sizes, thus assuming a parametric prior for the cis-eQTLs. The same parametric assumption is made by FUSION, since BSLMM assumes a normal mixture prior, combining Bayesian variable selection regression (BVSR)^17^ and linear mixed modeling (LMM).^18^ Given their parametric and linear assumptions, these tools fail to flexibly model the distributions of the genotypes and their relationship with gene expression.^19^ Some SNP and measured gene expression relationships can be best modeled mathematically with non-linear and non-parametric assumptions.^19,20^ Manor and Segal^20^ showed that by using simple non-linear modeling with the K nearest neighbor (KNN)^21^ algorithm, robust gene expression prediction can be achieved using just *cis*-eQTLs. Wang et al.^22^ found that a mixed model-based random forest (RF)^23^ (a non-linear model) has the potential to capture the non-linear relationships of *cis*-eQTLs and thus may improve gene expression imputation performance. Most recently, a method called TIGAR,^19^ which is based on a non-parametric Bayesian method called Dirichlet process regression,^24^ was shown to achieve a better imputation coefficient of determination (R^2^) than PrediXcan on simulation data where at least 1% of the cis-eQTLs are causal and true expression heritability is at most 0.2. TIGAR^19^ was also shown to impute expression for more genes than PrediXcan in a real dataset, thus corroborating the potential of using non-parametric and non-linear modeling of gene expression prediction in order to uncover more gene associations with complex traits.

Although several studies have shown that non-linear modeling of cis-eQTLs and gene expression can improve imputation performance,^19,20,22^ we sought to further explore the cross-population portability of both linear and non-linear transcriptome prediction in new cohorts. Generally, a large UK Biobank-based study has shown reduced accuracy in genetic prediction due to lack of diversity in training cohorts.^25^ More specifically, the importance of genetic ancestry diversity in gene expression prediction has also been corroborated by many recent studies, which have demonstrated that similarity in ancestries between the training and testing populations improves gene expression prediction.^26–29^ However, the replicability of these observations in new cohorts and how machine learning (ML) models perform across populations have not been adequately studied.

In this work, in order to further optimize gene expression imputation performance across global populations, we used two non-linear ML models, RF^23^ and KNN;^21^ a combination of both linear and non-linear ML models, support vector regression (SVR);^30^ and a linear ML model, EN, to predict gene expression from genotypes of SNPs within 1 Mb of each gene. We trained prediction models using genotype and blood monocyte transcriptome data from the Multi-Ethnic Study of Atherosclerosis (MESA)^26,31,32^ in self-identified African Americans (AFA, n = 233), Hispanic Americans (HIS, n = 352), European Americans (CAU, n = 578), as well as the combined cohort (ALL, n = 1,163). We tested MESA model performance on new genotype and whole-blood transcriptome data from participants enrolled in the Modeling the Epidemiology Transition Study (METS), which includes Ghanaians and African Americans (n = 76).^33,34^ We compared the ML models and showed gene prediction models were generally best in EN, with RF having the closest parallel performance. We corroborated previous findings that similarity in ancestry improves gene expression prediction accuracy. When we applied the ML models to transcriptome-wide association studies (TWASs) of lipid traits in MESA, we showed that RF models detect associations missed by EN. By integrating other ML modeling into PrediXcan and diversifying training populations to include more global ancestries, we may uncover new genes associated with complex traits that have not been previously studied.

## Material and methods

This study was approved by the Loyola University Chicago institutional review board (IRB) #210260091217 and Project #2014. Appropriate informed consent was obtained from human subjects.

### Genomic and transcriptomic training data

#### MESA

The MESA cohort is made up of 6,814 individuals recruited from 6 sites across the United States (Baltimore, MD; Chicago, IL; Forsyth County, NC; Los Angeles County, CA; northern Manhattan, NY; St. Paul, MN) and consists of 53% female and 47% male individuals between the ages of 45 and 84 years^31^ with the demographics approximately distributed as 38% CAU, 23% HIS, 28% AFA, and 11% Chinese American (CHN). From the whole cohort, RNA was extracted from CD14+ monocytes from 1,264 individuals across the three populations (AFA, HIS, CAU) and quantified on the Illumina Ref-8 BeadChip.^32^ Individuals with both genotype (dbGaP: phs000209.v13.p3) and expression data (GEO: GSE56045) included 234 AFA, 386 HIS, and 582 CAU. Illumina IDs were converted to Ensembl IDs using the RefSeq IDs from MESA and GENCODE^35^ version 18 (gtf and metadata files) to match Illumina IDs to Ensembl IDs. If there were multiple Illumina IDs corresponding to an Ensembl ID, the average of those values was used as the expression level.

#### MESA genotype data analysis and quality control

Genotype quality control and imputation were performed as previously described.^26^ To summarize, all MESA population genotypes were in genome build GRCh37/hg19. PLINK^36^ was used for quality control and cleaning of the genotype data. We removed SNPs with call rate < 99% or not in Hardy-Weinberg equilibrium (p < 0.00001), and linkage disequilibrium (LD) pruned the resulting SNPs by removing 1 SNP in a 50 SNP window if r^2^ > 0.3. We conducted identity by descent (IBD) analysis on the genotype data and removed one pair of related individuals (IBD > 0.05). The cleaned genotypes were merged with HapMAP populations (Yoruba in Ibadan, Nigeria [YRI]; Utah residents with Northern and Western European descent [CEU]; and East Asians from Beijing, China and Tokyo, Japan [ASN]), and principal component analysis was done both across and within populations using EIGENSTRAT.^37^ We used pre-LD-pruned variants and the Michigan Imputation Server and 1000 Genomes phase 3 v5 reference panel and Eagle v2.3 to impute genotypes in each of the MESA populations. The imputation reference populations were EUR for CAU and mixed population for AFA and HIS.^38–40^ Imputation results were first filtered by R^2^ < 0.8 and minor allele frequency (MAF) > 0.01, and ambiguous strand SNPs were removed. After filtering, 9,352,383 SNPs in AFA, 7,201,805 SNPs in HIS, and 5,559,636 SNPs in CAU were remaining for further analysis. After quality control, the final sample sizes used for the gene expression prediction model training are AFA = 233, HIS = 352, and CAU = 578. The final sample sizes used for downstream TWAS analysis are AFA = 1,188, HIS = 952, and CAU = 1,716.

#### MESA transcriptome data analysis and quality control

PEER factor (PF) analysis was performed on the expression data of each population using the peer R package.^41^ Mogil et al.^26^ showed that the true positive replication rate was similar for 10, 20, and 30 PEER factors. As such, in each of the MESA populations, we used 10 peer factors and 3 genotype principal components (Figure S1) to adjust for potential batch effects and experimental confounders in the measured gene expression data. Then, we quantile normalized adjusted expression levels for use in model building.

### Genomic and transcriptomic test data

#### METS

The METS cohort comprises 2,506 healthy individuals of African origin between the ages of 25 to 45 years, with approximately 500 (~50% male) from each of the five sites: Ghana; South Africa; Seychelles; Jamaica; and Chicago, IL, USA.^42^ Out of this cohort, 76 female individuals (37 Ghana and 39 Chicago, IL, USA) underwent genome-wide genotyping on the Illumina Infinium Multi-Ethnic AMR/AFR BeadChip and RNA sequencing (RNA-seq) from whole blood using the NuGEN mRNA-Seq with AnyDeplete Globin library preparation kit (Loyola IRB #210260091217). Single-end 50 bp RNA-seq was performed by the Duke University Sequencing and Genomic Technologies Shared Resource.

#### METS genotype data analysis and quality control

The METS genotype data are in genome build GRCh38/hg38. We performed all quality control using PLINK v1.90b4.4.^36^ We removed SNPs on non-autosomal chromosomes, below a call rate threshold of 0.01, or not in Hardy-Weinberg equilibrium (p < 0.00001). Prior to IBD and principal component analysis, we LD-pruned variants using PLINK indep-pairwise option at thresholds 50 5 0.3. Due to small sample size, we did not remove individuals based on cryptic relatedness. As such, we inferred the relationships of all pairs of individuals in our sample using KING^43^ package version 2.2.5. To account for the cryptic relatedness, we used the relationship inference from KING^43^ to calculate principal components (Figure S1) using the PC-Air^44^ tool in GENESIS^45^ package version 2.16.1. We performed METS genotype imputation on the Sanger Imputation service^40,46^ using the African Genome Resources reference panel and the pre-LD-pruned set of variants. After imputation, non-ambiguous strand SNPs in Hardy-Weinberg equilibrium (p > 0.05) with MAF > 0.05 and imputation R^2^ > 0.8 were retained, and the cleaned genotypes were lifted over to genome build GRCh37/hg19 for gene expression prediction analyses.

#### METS transcriptome data analysis and quality control

We used FASTQC^47^ to analyze RNA-seq quality and found 50 high-fidelity bases with no primers or over-represented sequences. We quantified gene expression using Salmon pseudoalignment,^48^ which estimates the transcripts per million (TPM) for each gene using a reference transcriptome without performing the time-consuming process of an actual alignment. We used only protein-coding genes as defined by GENCODE^35^ version 28 and removed genes with mean TPM < 0.01. The resulting expression data of all samples were quantile and rank normalized. We further adjusted for potential batch effects, experimental confounders, and population structure on all the sample expression levels with 10 PEER factors^41^ and 10 genotypic principal components (Figure S1). The resulting adjusted expression levels were used in downstream analysis.

#### Prediction models

In each of the MESA populations, we used the adjusted expression values for protein-coding genes and genotypes of SNPs within 1 Mb of each gene (i.e., in *cis*) to fit the models. Using nested cross-validation for EN, and 5-fold cross-validation for the other ML models, we calculate the R^2^ for how the model predicts on the held-out fold. We report the mean R^2^ over all 5 folds as our measure of model performance. R^2^ is defined as $1-{\sum \left(y_{0}-y_{p}\right)}^{2}/{\sum \left(y_{0}-{\bar{y}}_{0}\right)}^{2},$ where *y*_*o*_ is observed expression, *y*_*p*_ is predicted expression, and ${\bar{y}}_{0}$ is the mean of observed expression. Note that in this paper, R^2^ is not the square of the Pearson correlation coefficient. Instead, the coefficient of determination, R^2^ as defined above, can be negative and thus indicative of a poorly fit model. We used the fitted model to predict expression in METS. Model performance was evaluated by Spearman correlation (ρ) of the METS predicted and observed gene expression values defined by GENCODE^35^ version 28. Like prior studies, we considered ρ > 0.1 as significant.^11,26^ In our TWAS application of these models, we used the Bonferroni correction for the total number of genes tested across all four ML models (0.05/[5,279 + 3,651 + 3,772 + 2,601]) and thus considered (p < 3.3 × 10^−6^) to be significant.

#### EN

We used the glmnet R package^49^ to implement EN with the alpha parameter set at 0.5, which has previously been shown to perform optimally for predicting gene expression.^10^ Alpha is the mixing parameter of EN used to achieve the combination effect of lasso (alpha = 1) and ridge (alpha = 0) penalties. For every single gene, we carried out nested cross-validation of the EN model as follows: first, training data were split into roughly five equal parts; second, for each held-out fold, 10-fold cross-validation was performed on the remaining four folds to minimize the lambda parameter, and the model with the minimal lambda was used to predict on the held-out fold to determine the R^2^. Lambda is a tuning parameter that controls the overall strength of the EN penalty in each gene model. After going through each of the five folds, we used the average R^2^ as our measure of model performance. The trained models with minimal lambda were used to predict expression in the test data.^26^

#### RF

We used the scikit-learn Python package version 0.21.2^50^ (Python version 3.7.3) to implement RF regression, and all the hyperparameters in the regressor were set to default except for the *n_estimators* hyperparameter (which is the number of trees in the forest). For every single gene, via 5-fold cross-validation, we conducted a grid search of the best *n_estimators* hyperparameter ranging from 50 to 500, inclusive, that yields the highest cross-validated regression R^2^. The range of trees used in the grid search was informed by our preliminary analysis result as shown in Figure S2. Subsequently, for every gene, we used the resulting best *n_estimators* hyperparameter to fit the RF regressor model and predict expression in the test data. See Table S1 for the optimum number of trees for each gene across training populations.

#### KNN

We used the scikit-learn Python package version 0.21.2^50^ (Python version 3.7.3) to implement KNN regression. The hyperparameters were set to default except for *n_neighbors* (which is the number of neighbors [*k*] to use), *weights* (which is a weight function used in the prediction), and *P* (which is the power parameter for the Minkowski metric). We used two of the *weights* function parameters, namely “uniform” (wherein all points in each neighborhood are weighted equally) and “distance” (wherein all points in each neighborhood are weighted by the inverse of their distance). For every gene, via 5-fold cross-validation, we conducted a grid search of the best three hyperparameter combinations that yield the highest cross-validated regression R^2^. The three hyperparameter combinations were drawn from *k* (odd numbers between 3 and 31 inclusive), *weights* (uniform and distance), and *P* (1, 2, 3). Subsequently, for every gene, we used the resulting best hyperparameter combination to fit the KNN regressor model and predict expression in test data. See Table S2 for the optimum hyperparameter combinations for each gene across training populations.

#### SVR

We used the scikit-learn Python package version 0.21.2^50^ (Python version 3.7.3) to implement SVR. We set all parameters to default except for the followings: *gamma* (controls the bias-variance trade-off of each gene model, where small values mean far-reaching radius of influence while large values mean close radius of influence. We set it to “scale” because we want the *gamma* value to be determined by the variance and number of predictors in each gene model), *kernel* (which is the type of mathematical function used to transform data in the model), *degree* (which is specifically for the degree of the polynomial kernel function), and *C* (which is the penalty for error term). For every gene, via 5-fold cross-validation, we conducted a grid search of the best three hyperparameter combinations that yield the highest cross-validated regression R^2^. The three hyperparameter combinations were drawn from *kernel* (“linear,” “poly,” “rbf,” “sigmoid”), *degree* (2, 3, 4, 5, 6, 7), and *C* (0.0001, 0.0005, 0.001, 0.005, 0.01, 0.05, 0.1, 0.5, 1.0, 1.5, 2.0). Specifically, the kernels are divided into two groups: linear kernels, which includes only “linear,” and non-linear kernels which include “poly,” “rbf,” and “sigmoid.” Thus, the kernel used determines if the SVR model is a linear or non-linear model. Subsequently, for every gene, we used the resulting best hyperparameter combination to fit the SVR regressor model and predict expression in test data. The number of gene models with R^2^ > 0.01 built with different kernels is distributed as follows: AFA = 340, 1,243, 501, 564; CAU = 1,065, 1,269, 577, 476; HIS = 595, 1,210, 608, 643; ALL = 1,600, 1,288, 653, 231; for “linear,” “poly,” “rbf,” and “sigmoid” kernels, respectively. See Table S3 for the optimum hyperparameter combinations for each gene across training populations.

#### Model standardization

In addition to our user-defined grid searches described above, we also compared predictive performance among all the four ML models by implementing them in the same package with standardized hyperparameter tuning. We implemented all the tested ML models (EN, RF, SVR, and KNN) with scikit-learn Python package version 0.21.2^50^ (Python version 3.7.3) and used Hyperopt^51^ version 0.2.4 to standardize the hyperparameter tuning across the ML methods. Specifically, we fixed the maximum number of evaluations (max_evals = 30) for the ML models. The choice of setting the maximum evaluations to thirty is to reduce computational time, especially for RF, which takes a longer time to run. Thus, for EN versus KNN, and EN versus SVR, like in grid search above, we built models for all protein-coding genes in chromosomes 1–22, while for EN versus RF, we focused only on chromosome 22. See Figure S3 for the model comparisons.

## Results

### EN outperforms ML models for cross-validated gene expression prediction

**Figure 1.**
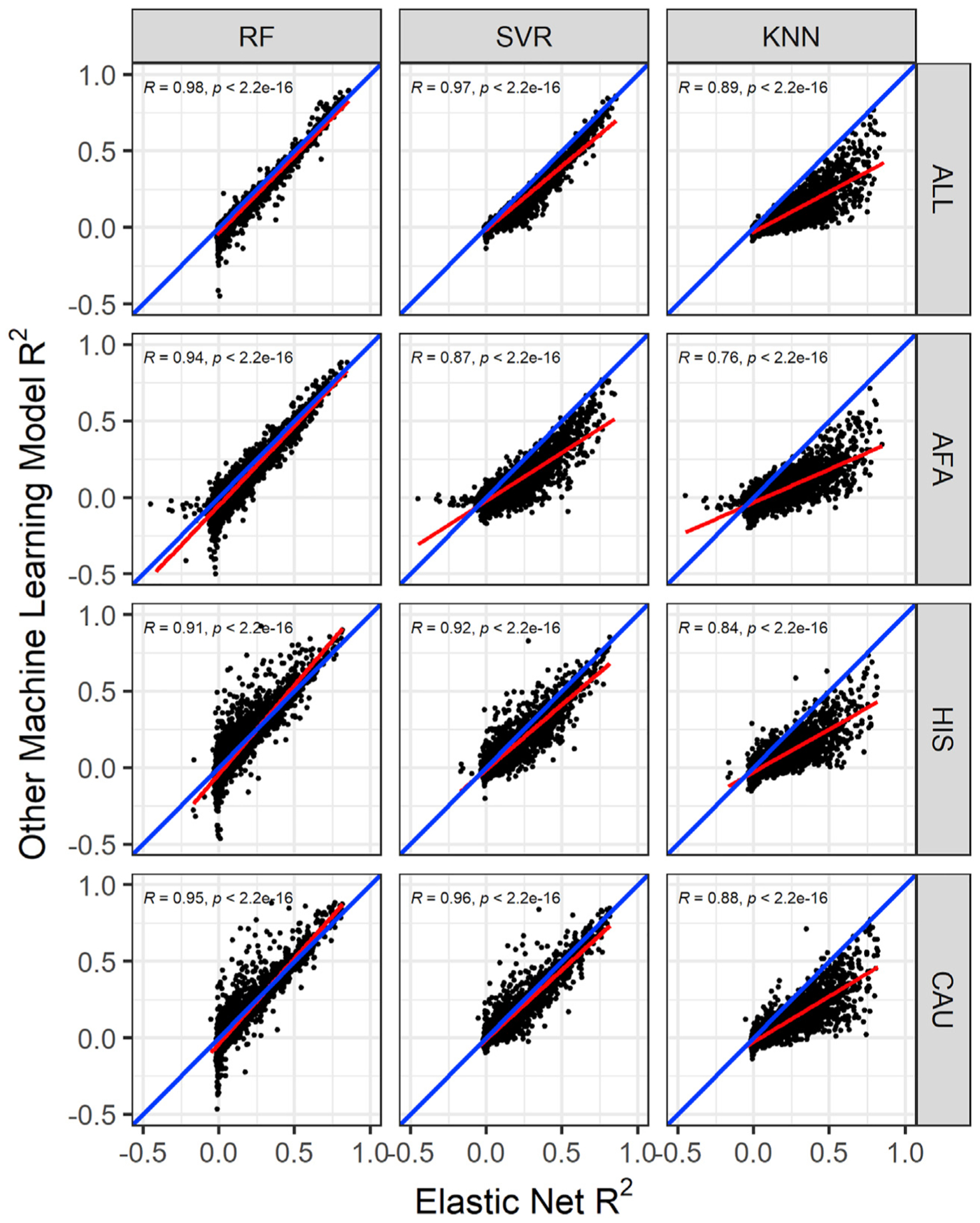
Comparison of the cross-validated gene expression prediction performance in the MESA cohort Gene expression prediction R^2^ between elastic net (EN) and other machine learning (ML) models across MESA populations. The linear regression fit is shown by the red line, and the identity line (slope = 1) is blue in each plot. In the ALL cohort (combination of AFA, HIS, and CAU populations), the RF model has 9,621 genes, while the SVR and KNN models have 9,622 genes in common with EN. Pearson correlations (*R*) between EN performance and random forest (RF), support vector regression (SVR), and K nearest neighbor (KNN) are shown in each plot. All correlations are significant (p < 2.2e−16). In the AFA cohort, the overlapping genes between models are RF versus EN = 9,608, SVR and KNN versus EN = 9,609. In the HIS cohort, the other ML models each have 9,499 genes in common with EN. In the CAU cohort, ML models have 9,499 genes in common with EN. EN generally outperformed RF, SVR, and KNN, except for some genes where RF outperforms EN, particularly in the HIS and CAU populations.

We sought to determine if untested ML models could improve SNP-based imputation of the transcriptome across populations compared to the parametric EN models currently used in PrediXcan.^11^ We trained each of the ML algorithms—RF, SVR, and KNN—using genotype and blood monocyte transcriptome data from each population in the MESA. The training samples in the MESA populations are distributed as AFA (n = 233), CAU (n = 578), and HIS (n = 352). To have a larger sample size, we also combined the genotype and transcriptome of the MESA populations (AFA, HIS, CAU) into the ALL cohort (n = 1,163). Standard quality control analysis was done on the genotype and expression data to adjust for population structure and potential experimental confounders (see Material and methods). Using each of the MESA populations and ALL, we then performed model training through 5-fold cross validation of RF, SVR, and KNN and nested cross-validation of EN by using SNPs within 1 Mb of each gene to predict its expression level. We used the R^2^ between predicted and observed expression as our measure of model performance (see Material and methods). We found that across all the populations and prediction algorithms, *ERAP2* (MIM: 609497), *HLA-C* (MIM: 142840), *HLA-DRB1* (MIM: 142857), *CHURC1* (MIM: 608577), *RAD51* (MIM: 179617), and *SNAP29* (MIM: 604202) have R^2^ > 0.5. We also found that EN usually outperformed the ML models, but RF outperformed EN on some gene models, especially those trained in HIS and CAU (Figures 1 and S4). This suggests that different prediction algorithms may be potentially more robust for different training populations.

To better ensure our comparison of the four ML models was not affected by our chosen software packages and grid search spaces, we also compared standardized models using Hyperopt^51^ (see Material and methods). Hyperopt is a Python library that standardizes model selection and hyperparameter optimization.^51^ Gene expression prediction model performance obtained from our implementation of the Hyperopt^51^ standardization approach maintained the same trend of EN outperforming the other three tested ML models (Figure S3). Thus, we use our grid search optimization approach in the ML model results described in the rest of this paper.

Focusing only on the model training built in the ALL cohort, the model building converged and completed for 9,623 genes in RF, SVR, and KNN and 9,622 in EN. The 9,622 genes in EN models are also in SVR and KNN, while 9,621 are in RF. The average R^2^ for each of the prediction algorithms is EN = 0.0733, SVR = 0.0476, RF = 0.0409, and KNN = 0.0103. *TACSTD2*, *RNF150*, *HLA-DRB5*, *HLA-DRB1*, and *CHURC1* genes have R^2^ > 0.8 across EN, RF, and SVR models, while all genes in the KNN model have R^2^ < 0.8. Overall, EN significantly outperformed all ML models, as shown in Figure 1 and Table 1. Focusing on the overlapping genes with R^2^ > 0.01 (EN versus SVR = 3,736, EN versus RF = 3,635, EN versus KNN = 2,598), EN performed better on approximately 99%, 97%, and 93% of the overlapping genes than KNN, SVR, and RF, respectively. Table 2 shows the number of genes that have models in each of the prediction algorithms at different R^2^ thresholds. EN had the most gene models compared to the other ML methods across all thresholds. However, at R^2^ > 0.5, RF has almost same number of gene models as EN (RF = 194, EN = 222), distantly followed by SVR, while KNN has just 28 genes. This clearly shows that EN, RF, and SVR models have generally good performance for most of the highly predictable genes. The same comparison trend is generally observed in the models trained with AFA, CAU, and HIS (Tables S4–S6). However, while mean predictive performance was higher for EN across populations (Table 1), we observed that RF outperformed EN for some genes, especially in HIS- and CAU-trained data (Figure 1). This suggests integrating both EN and RF models into transcriptome prediction may be useful. Next, we sought to determine how our models performed in an independent test cohort.

### Similarity in ancestry improves prediction performance across prediction models

Recent studies using EN have observed that similarity in training and testing population improves prediction performance.^26–29^ In order to see if the same observation replicated with additional ML algorithms, we used new genotype and whole-blood transcriptome data from 76 African American individuals in Chicago, Illinois (USA) and Africans in Ghana enrolled in METS as a replication cohort.^34,42^ We performed standard quality control and adjusted for potential confounders in the METS genotype and transcriptome data (see Material and methods). We predicted gene expression in the METS cohort using only gene models with cross-validated R^2^ > 0.01 in each of the prediction algorithms trained with the MESA cohort. Specifically, we tested models trained in each of the MESA populations (AFA = 233, HIS = 352, CAU = 578) and the combined population (ALL = 1,163). To accommodate for any effect sample size may have in our study, we also used the combination of AFA and HIS populations (AFHI = 585), which is a similar sample size as CAU, to train the prediction algorithms. Both AFA and HIS contain recent African admixture and thus share more genetic ancestries with our test cohort (METS) than CAU (Figure S5). To determine how accurate the prediction algorithms trained in MESA are in METS, we computed the Spearman correlation (ρ) between the METS predicted expression values and METS measured expression values.

To evaluate the prediction performance of the training MESA population in METS, for each of the prediction algorithm methods, we calculated the mean ρ for genes predicted in all 5 of the populations (Table 3). Across the training populations, the mean ρ in METS is highest when using AFHI-trained models for all the prediction algorithms. As shown in Table 3, across all the tested prediction algorithms, the training populations comprising individuals of recent African ancestries (AFA, HIS, AFHI, ALL) significantly outperformed the training population comprising only individuals of European descent (CAU) (Welch’s t test, all algorithm p values < 0.0210, except for KNN, where HIS versus CAU p value = 0.1226). This shows that prediction performance is highest when the genetic distance between the training population and testing population are closest, regardless of the prediction algorithm used. Also, larger sample size improves prediction performance but not as much as when majority of the individuals in the training set share similar ancestries with those in the test set (i.e., AFHI-trained models perform the same as ALL-trained models) (Welch’s t test, all algorithm p values > 0.6360) (Table 3). If larger sample size were the main factor to improve prediction performance, we would expect the average ρ to be significantly higher in ALL. However, we see that average ρ in the ALL is less than in the AFHI, even though AFHI has lower sample size. More so, the ALL-trained models’ average ρ were not significantly better than AFA-trained models (Welch’s t test p values, EN = 0.5053, RF = 0.3782, SVR = 0.0424, KNN = 0.5391). AFA has the lowest sample size and closest ancestry similarity to METS across the training MESA populations. Thus, this highlights the importance of similarity in ancestry at improving prediction performance.

**Figure 2.**
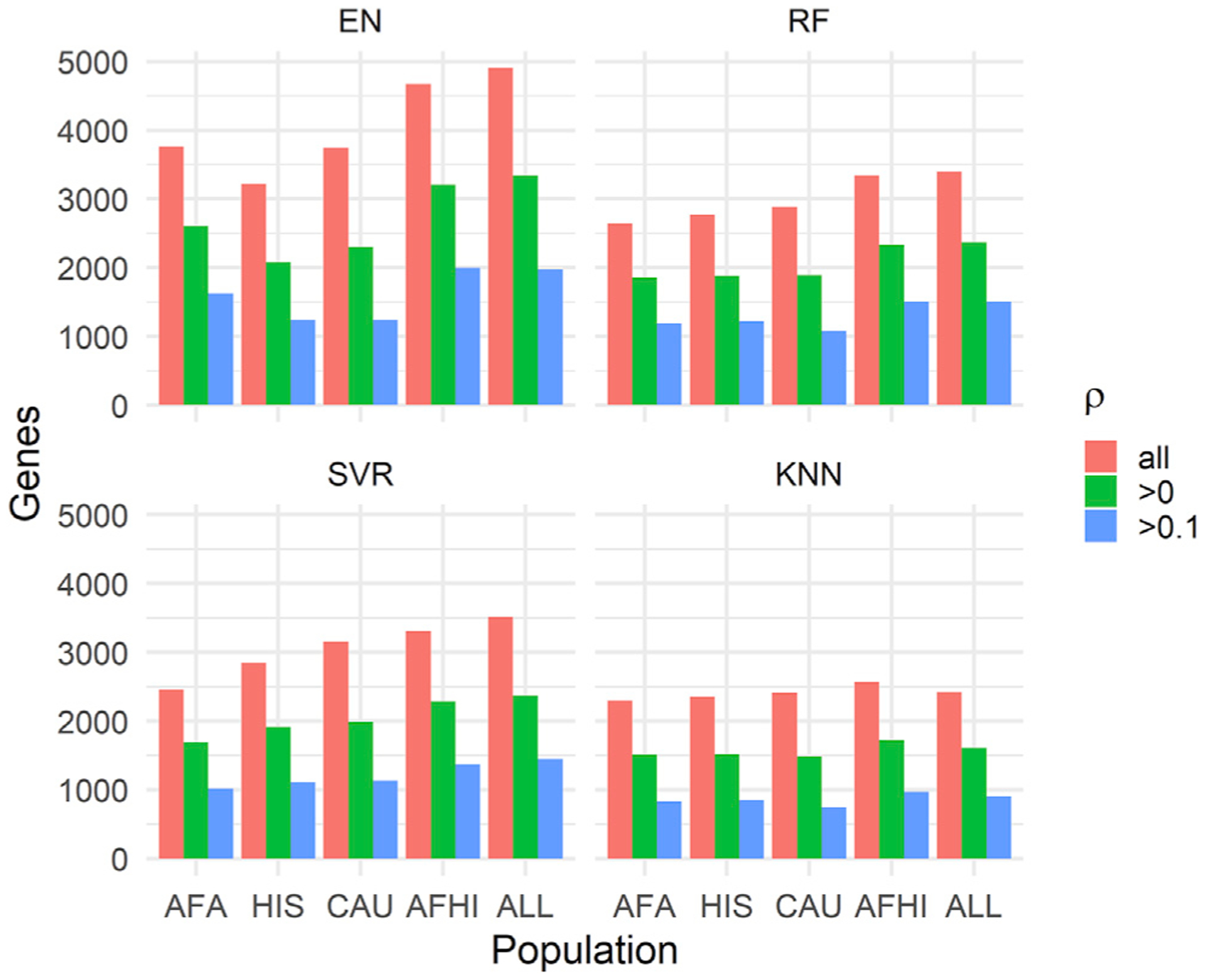
Number of predicted genes in METS after filtering by ρ The MESA population used to train each set of models is shown on the x axis, and the number of genes with predicted expression values in METS is shown on the y axis. ρ is the Spearman correlation between predicted and observed gene expression in METS.

When we examine all prediction results in METS, the number of genes we were able to predict gene expression values for varied across algorithms and populations (Figure 2). The gene models trained with the ALL cohort predicted gene expression values for more genes than the other training populations across all prediction algorithms. This is probably because the ALL cohort had the largest sample size. In fact, the number of genes captured decreases from ALL to AFA as the sample size decreases, with the exception of EN trained on HIS. Interestingly though, when we filter by ρ > 0.1, EN trained on AFA captures more genes (1,622) than HIS (1,238) and CAU (1,238), while RF trained on HIS (1,219) and AFA (1,190) each capture more genes than CAU (1,078), despite CAU having a larger sample size than AFA and HIS. This again shows the importance of similarity in ancestry between training and testing population for gene expression prediction. The models trained with AFHI and ALL cohorts capture more genes than AFA, most probably because of their larger sample size and the fact that they also contain the AFA cohort. Therefore, although larger sample size is important in prediction performance, it is paramount that individuals in the training population have similar ancestry with the testing population.

### EN-trained models outperform ML models in test cohort

**Figure 3.**
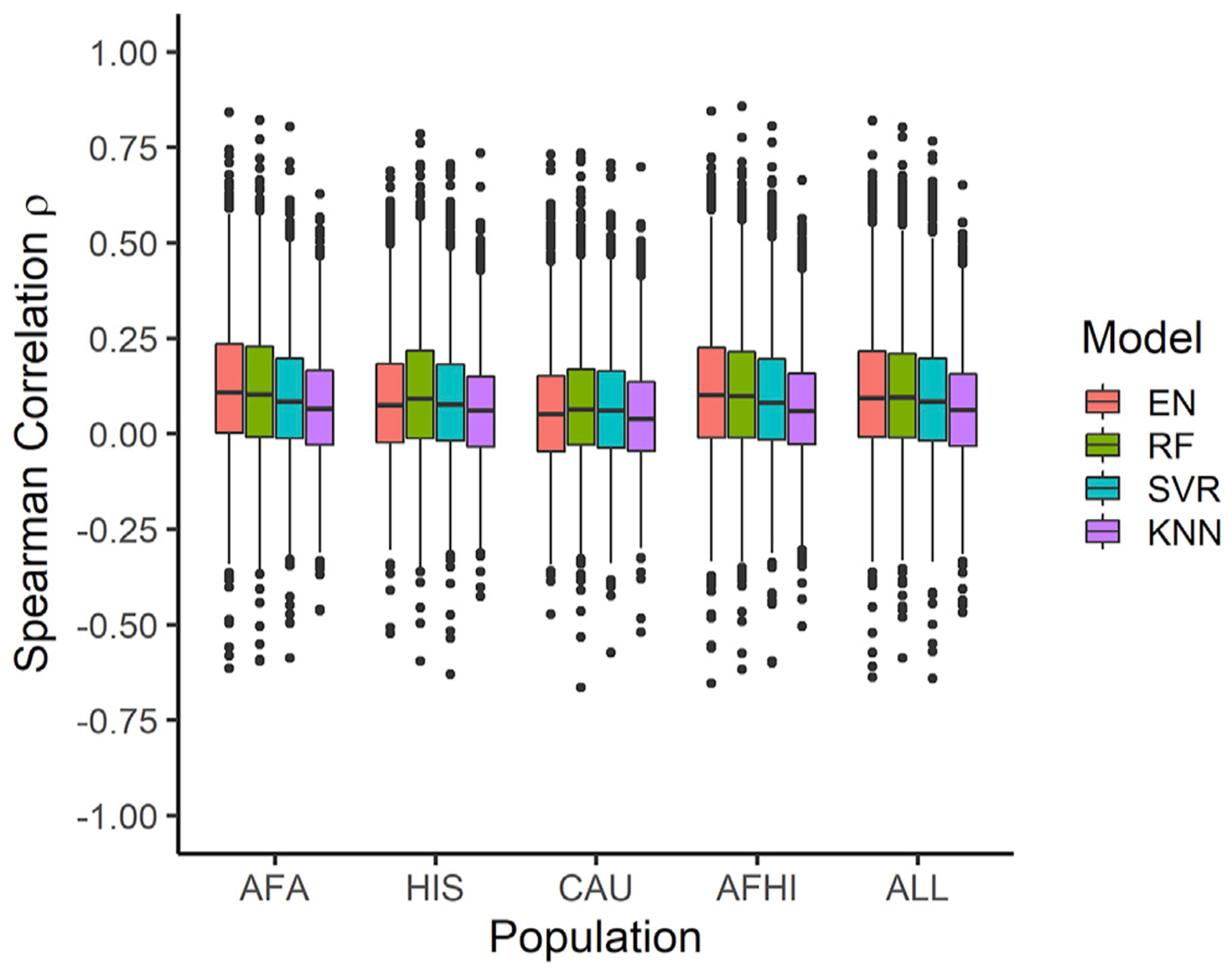
Prediction performance of models trained in MESA populations and tested in METS We predicted expression in METS using only gene models with R^2^ > 0.01. The MESA population used to train each set of models is shown on the x axis, and the Spearman correlation between predicted and observed gene expression in METS is shown on the y axis. For each training population, only gene intersects of all prediction algorithms are shown in the plot. For example, in AFA, all gene intersects of EN, RF, SVR, and KNN are plotted.

**Figure 4.**
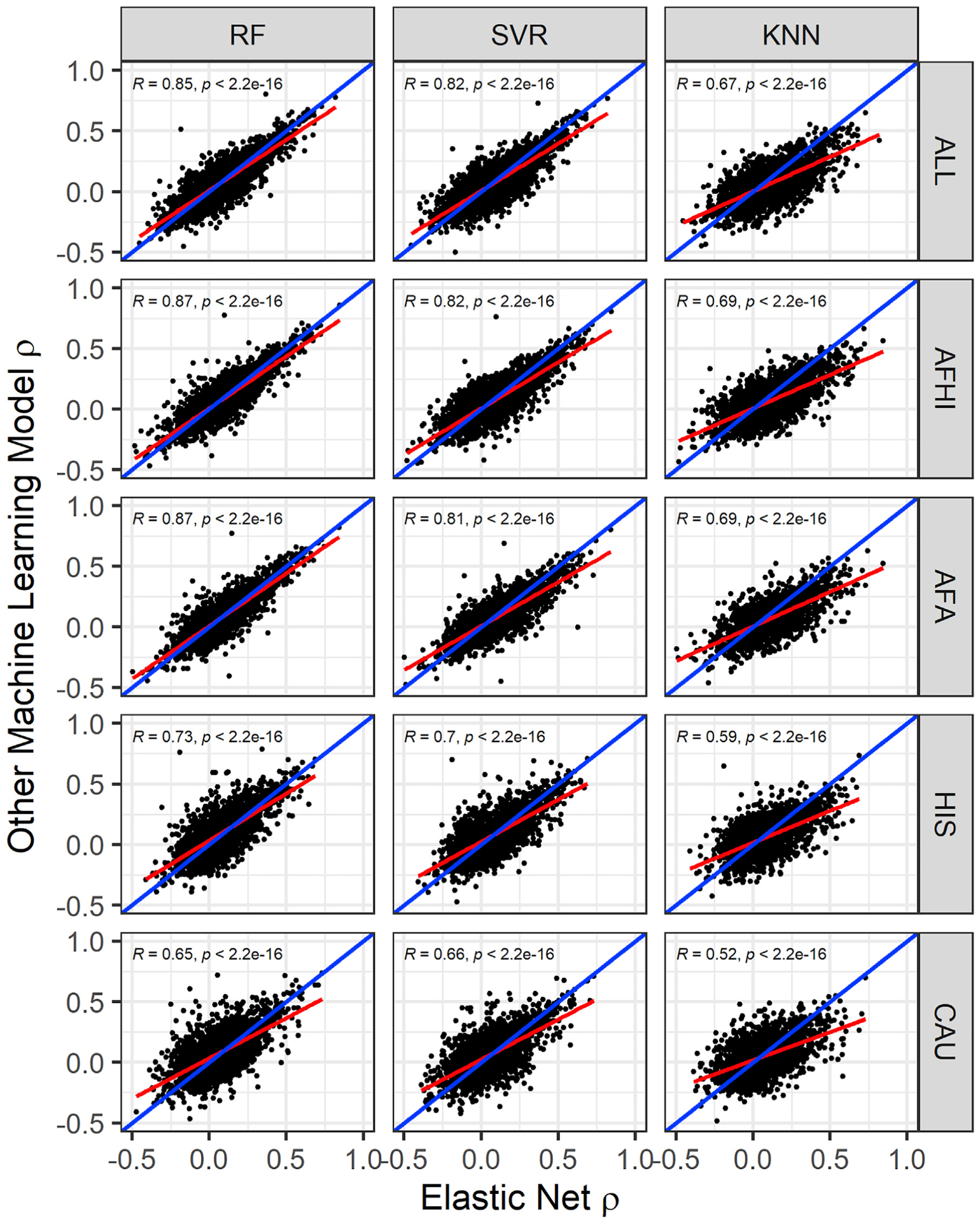
Comparison of algorithm test prediction performance in METS from models trained in MESA Prediction performance ρ (Spearman correlation between predicted and observed gene expression in METS) for each gene in each other ML model versus EN is shown. The linear regression fit is shown by the red line, and identity line (slope = 1) is blue in each plot. Pearson correlations (*R*) between performance are shown in each plot (all p < 2.2e-16). In the ALL cohort, the number of genes that overlap are EN versus RF = 3,378, EN versus SVR = 3,477, and EN versus KNN = 2,414. In the AFHI cohort, the number of genes that overlap are EN versus RF = 3,269, EN versus SVR = 3,166, and EN versus KNN = 2,482. In the AFA cohort, the number of genes that overlap are EN versus RF = 2,414, EN versus SVR = 2,125, and EN versus KNN = 1,894. In the HIS cohort, the number of genes that overlap are EN versus RF = 2,374, EN versus SVR = 2,342, and EN versus KNN = 1,995. In the CAU cohort, the number of genes that overlap are EN versus RF = 2,686, EN versus SVR = 2,855, and EN versus KNN = 2,255.

EN predicts gene expression values in METS for more genes than RF, SVR, and KNN (Figure 2). When all genes predicted in METS by all 4 of the prediction algorithms for each training population are compared, mean prediction performance (ρ) is significantly highest for RF-trained models in the HIS and CAU populations, while mean prediction performance is highest for EN-trained models in the AFA, AFHI, and ALL populations (Figure 3; Table 4). Furthermore, when we compare test prediction performance of each of the ML algorithms against EN on the genes they both can predict (intersection) for each training population, EN performs best regardless of training population except in HIS and CAU, where mean prediction performance was again better in RF than EN (Figures 4 and S6; Table 5). Focusing only on ALL-trained models, the number of overlapping genes between EN and the other algorithms are RF = 1,198, SVR = 1,141, and KNN = 676.

Although generally EN outperforms the other algorithms, we observe that all the genes in each of the algorithms did not overlap with those in EN even though they captured fewer genes than EN (Table 6). That is, these algorithms have significant performance (ρ > 0.1) on some genes that EN does not, and vice versa. To probe further into the algorithm pairs, we counted the genes unique to each algorithm (Table 6). Expectedly, EN captures 778 unique genes; however, the few unique genes (<310) captured by each of RF, SVR, and KNN suggest that prediction performance in test cohorts may be improved by combining gene models from EN and these other algorithms. Focusing only on the RF and EN sets of unique genes, we found that the average normalized expression levels were slightly higher in the RF group (mean = 0.0318) than the EN group (mean = 0.0291) (Welch’s t test p value = 0.0014). Additionally, the average variance in the normalized expression levels was slightly higher in the RF group (0.678) than the EN group (0.639) (Welch’s t test p value = 0.019). Since the magnitude of these differences is not large, it is unlikely variation in the expression levels is the reason these genes are captured only by the RF algorithm. Moreover, model performance and, by extension, ability to capture unique genes is not driven by or correlated with expression levels (Figures S7 and S8). In addition, upon performing principal component analysis of expression levels, we found that the genes did not cluster by prediction algorithm (Figure S9).

### EN and ML models identify the same gene in lipid TWASs

**Figure 5.**
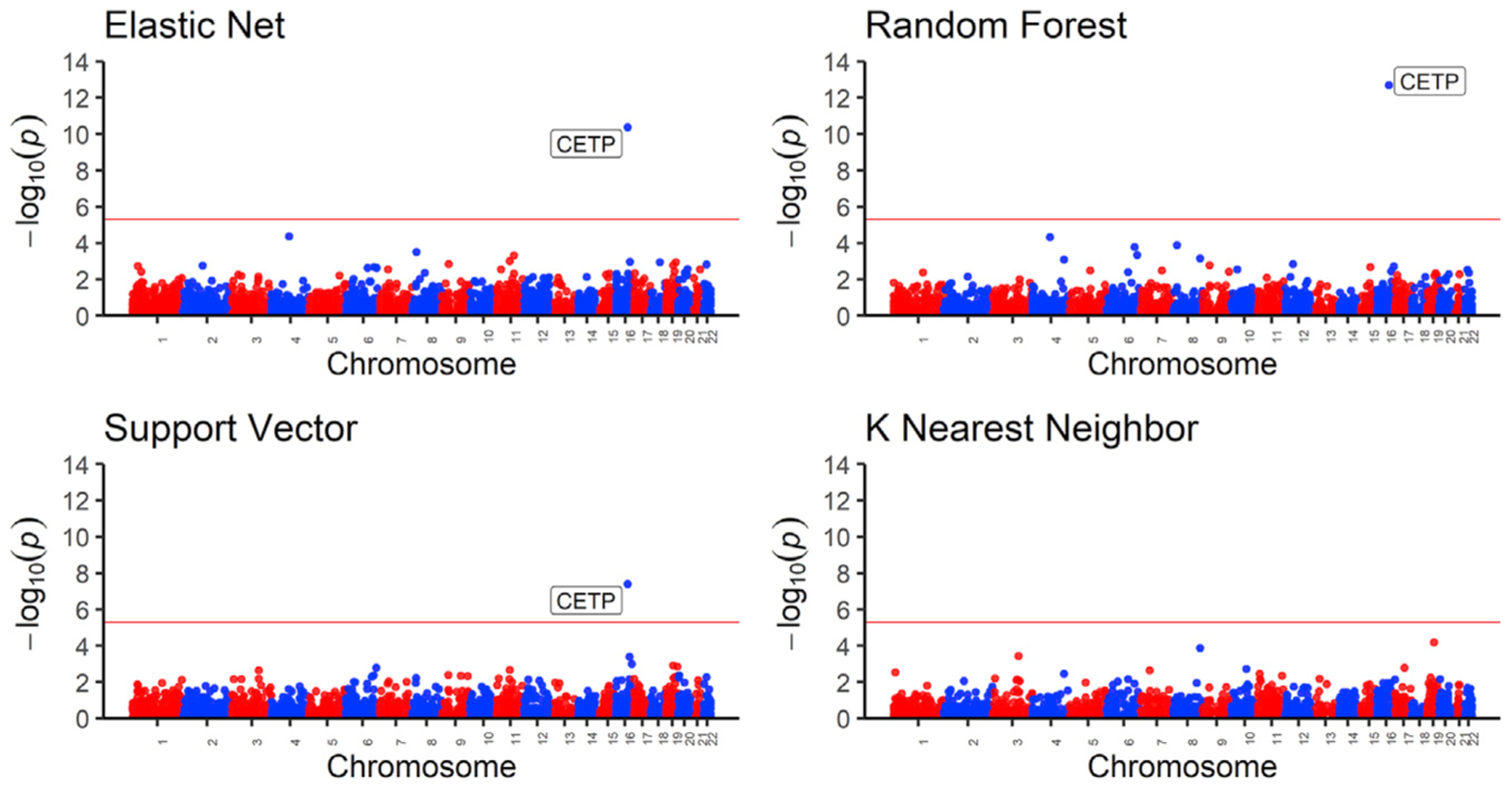
High-density lipoprotein (HDL) transcriptome-wide association study (TWAS) results Manhattan plot of the gene p values from the TWAS between HDL values and predicted gene expression. Using models trained in MESA ALL cohort, we predicted gene expression in MESA (n = 3,856) genotype data comprising individuals not used in the model training with HDL phenotype data and then carried out in TWAS. Genome-wide significance (p < 3.3 × 10^−6^) is shown by the red line in the plots. The x axis is ordered from chromosomes 1 to 22 (left to right).

**Figure 6.**
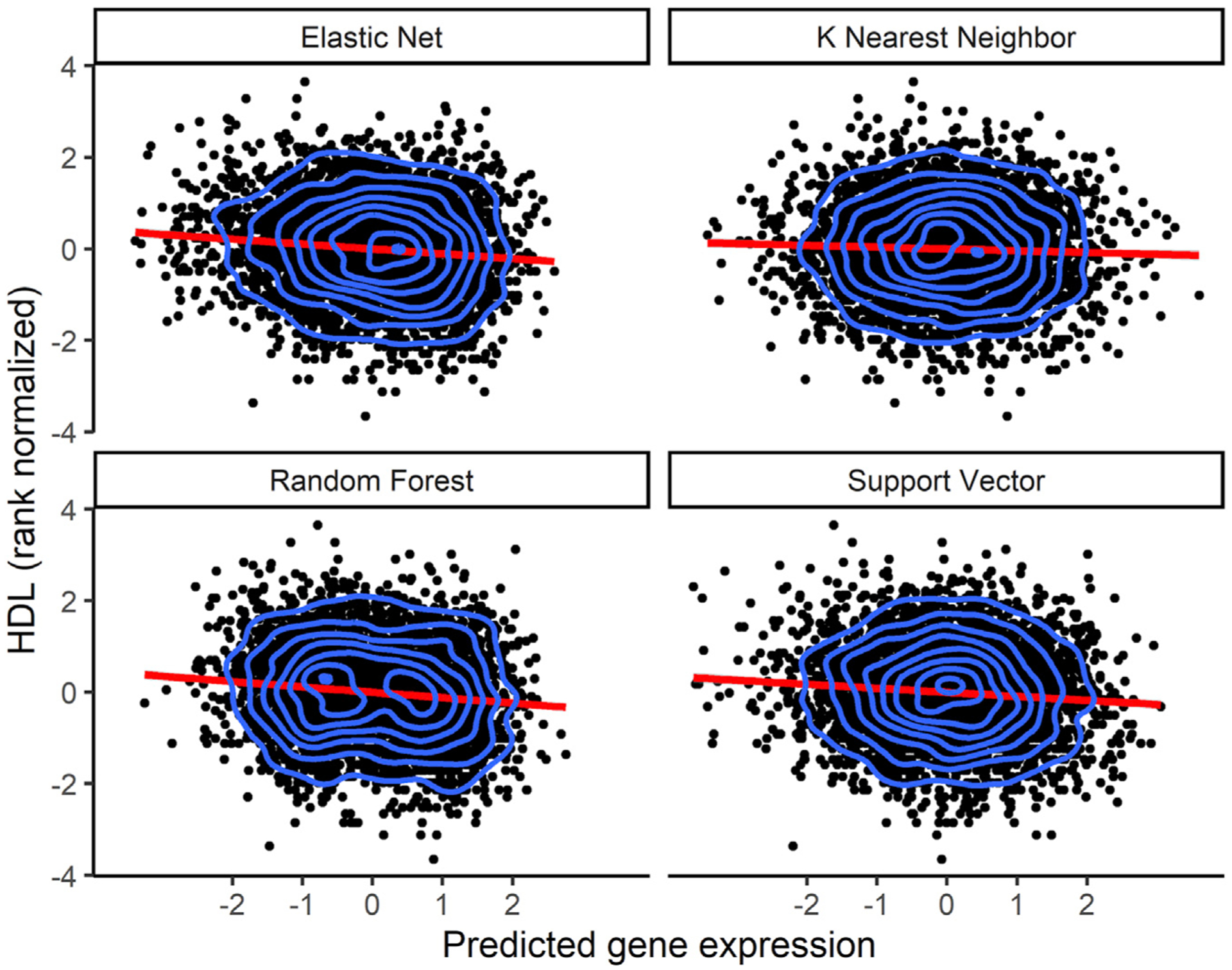
Increased HDL levels correlate with decreased *CETP* predicted expression Direction of effect of the *CETP* gene on HDL levels. Using models trained in the MESA ALL cohort, we predicted gene expression in MESA (n = 3,856) genotype data comprising individuals not used in the model training with HDL phenotype data. Each point in the plot represents an individual. The linear regression fit is shown by the red line in each plot. The blue contour lines from two-dimensional kernel density estimation help visualize where the points are concentrated. Although KNN is shown here, the *CETP* gene HDL TWAS with KNN was not genome-wide significant (p = 0.016). The EN (p = 4.1 × 10^−11^), RF (p = 2.1 × 10^−13^), and SVR (3.9 × 10^−8^) models were genome-wide significant.

**Figure 7.**
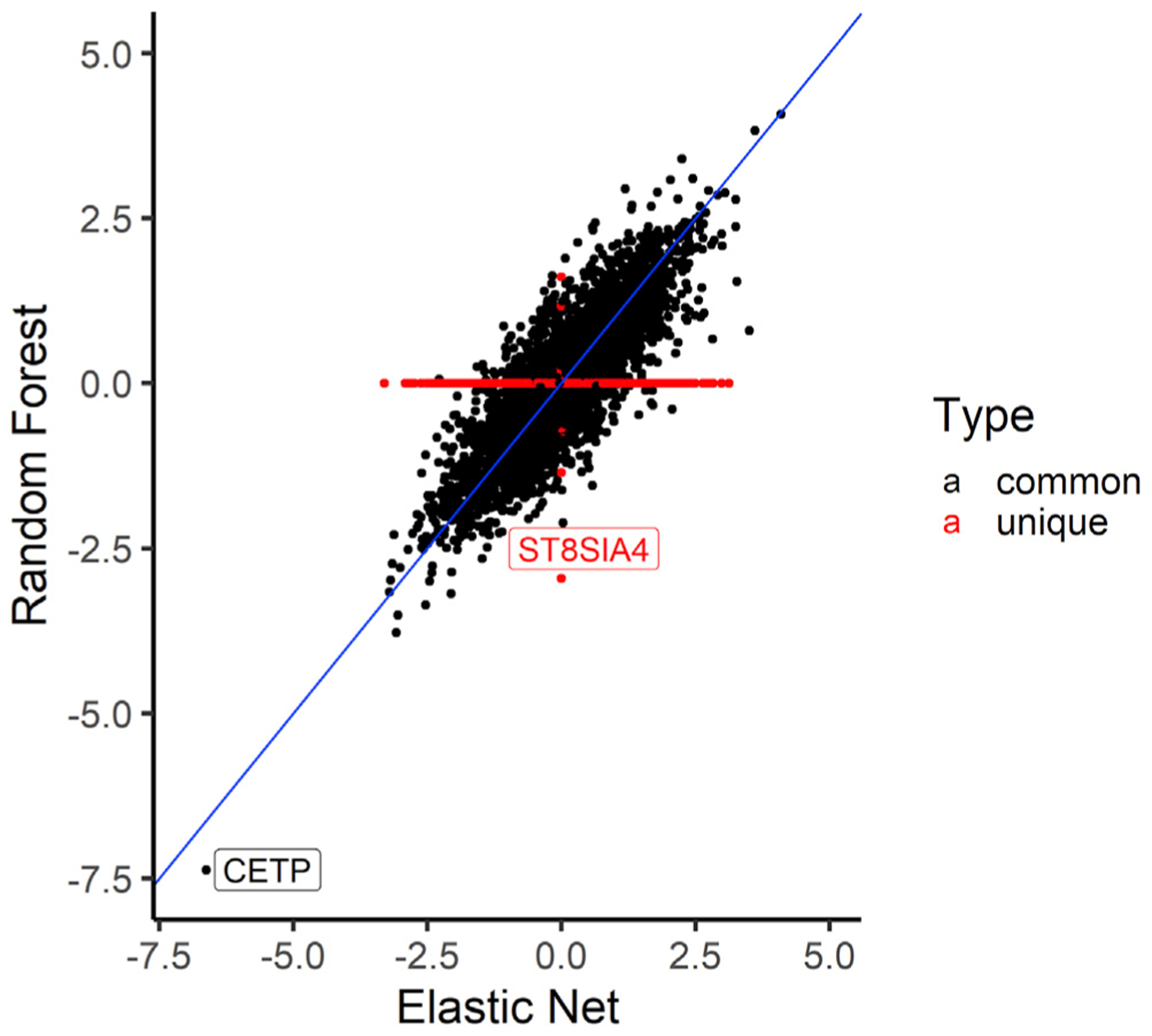
Comparison of the HDL association t-statistics from RF and EN models trained in the MESA ALL cohort Comparison of RF and EN t-statistics from the TWAS of HDL and predicted transcriptome in MESA individuals not used for imputation model building. Each dot in plot represents the t-statistic value of a gene from the HDL TWAS, while the identity line (slope = 1) is shown in blue. We see that the t-statistic values are similar between RF and EN except for genes that are unique in each algorithm shown as red dots in the plot. *CETP* is strongly associated with HDL levels using both EN- and RF-trained models. RF-trained models revealed the unique gene *ST8SIA4* (no prediction model in EN) may be potentially associated with HDL levels (p = 4.3 × 10^−3^).

To evaluate the biological importance of the prediction algorithms in identifying significant genes associated with traits, we carried out TWASs on high-density lipoprotein (HDL) levels. In our analysis, we used a genotype dataset from the MESA cohort (n = 3,856), comprising individuals from the populations that were not used in building any of the imputation models and in which we have corresponding lipid phenotype data (AFA = 1,188, HIS = 952, and CAU = 1,716). The genotype data were cleaned using standard quality-control procedures (see Material and methods). We used the ALL-trained imputation gene models (genes with cross-validated R^2^ > 0.01) from each algorithm to impute transcriptome levels from the MESA genotypes. We adjusted the predicted transcriptome levels for population structure using the first 3 genotype principal components (Figure S1) and rank normalized the HDL levels. Using the adjusted predicted transcriptome levels and normalized HDL data, we conducted association tests using linear regression. Interestingly, all tested prediction algorithms except KNN identified a significant association (p < 3.3 × 10^−6^) for the cholesteryl ester transfer protein, plasma gene (*CETP* [MIM: 118470]) (Figures 5 and S10). The lack of association with HDL for all gene-expression values predicted from KNN-trained models is consistent with our earlier results in this paper that KNN is worse at imputing transcriptome levels compared to the other algorithms. The directions of effect of *CETP* transcriptome levels as predicted by EN, RF, and SVR are the same (Figure 6). An increase in predicted *CETP* expression is associated with decreased HDL levels across EN, RF, and SVR. The ability of the three algorithms to identify the same significant hit underscores their effectiveness at imputing gene expression (*CETP* R^2^: EN = 0.0917, RF = 0.0772, SVR = 0.0539). Consequently, wecompared EN and RF t-statistic values from the association tests between HDL and predicted gene expression. We found that both EN and RF t-statistic values were almost parallel for the genes they have in common, thus corroborating the observed similar performance on their common genes from our previous results (Figures 1 and 3). In the EN TWAS, 5,279 genes were tested for association with HDL. In the RF TWAS, 16 unique genes that were not present in the EN TWAS were tested for association with HDL (Figure 7). Among the RF unique genes, we found a potential gene, *ST8SIA4* (MIM: 602547), that may be associated with normalized HDL (p = 3.192 × 10^−3^) but was missed by EN (*ST8SIA4* R^2^: EN = −0.0005, RF = 0.0100) (Figure 7). Although the association did not pass the Bonferroni correction to be genome-wide significant, this discovery is consistent with our previous results, wherein we found that although EN has many genes in common with RF in their imputation models, the RF algorithm generated some unique gene models (Table 6). Thus, by combining EN and RF models in gene expression imputation and subsequent TWAS analysis, we may uncover more and new significant gene-trait associations. Note, however, that by combining EN and RF models, we are not significantly changing the number of tests performed. Depending on predictive performance inclusion threshold, adding RF expression prediction models may increase the number of tests by up to 16% (Table 6), which does not dramatically change the Bonferroni correction threshold.

## Discussion

In this paper, we explored the potential of using RF, SVR, and KNN to further improve gene expression prediction performance across global populations in comparison to EN modeling, which is currently used in PrediXcan.^11^ To accomplish this, we trained each of the prediction models with genotype and transcriptome data from the MESA cohort on 9,623 protein-coding genes and compared their cross-validated imputation performance (R^2^). Although almost paralleled by RF and SVR, we found EN generally outperformed the other tested ML models. This is consistent with a recent study where it was shown that the genome-wide polygenic risk score method based on simple linear additive effects of genetic factors outperformed ML models in genetic prediction of cardiovascular disease risk.^52^ However, in our study, we found that when the prediction models are trained within each of the MESA populations, RF sometimes outperformed EN, specifically on HIS and CAU data (Figures 1 and 3; Tables 1 and 4). This suggests potential robustness and reduced variability of RF imputation performance across global populations.

We further tested the MESA-trained models on genotype and transcriptome data from African-origin individuals in the METS cohort. We show that models trained with the cohorts (AFA, HIS, AFHI, ALL) comprising individuals similar in ancestries with METS have better prediction performance than the models trained with individuals (CAU) of no recent African ancestries (Table 3; Figure 3). Thus, as demonstrated in several recent studies,^26–29^ here we also show similarity in ancestries between training and testing populations improves prediction performance. Notably, we found that the improvement in prediction due to ancestry similarity is consistent within all tested prediction algorithms, further underscoring the huge importance of diverse ancestries in genetic studies.

In the application of the MESA-trained models to the METS cohort, we further compared the prediction performance of EN against the other ML models. Although EN consistently outperformed the other tested models (which further corroborates the cross-validated performance results), we found gene models that are unique to each prediction algorithm (Table 6). Further analysis suggests there is nothing strikingly unusual in the expression levels of these groups of genes (Figures S7–S9). Therefore, it is unlikely variation in the expression levels is the reason these genes are captured only by one algorithm over another.

We applied the trained models on out-of-sample MESA genotype data with corresponding HDL phenotype values. All tested prediction models except for KNN identified the gene *CETP* to be significantly associated with HDL. As seen in a recent study on lipid traits,^53^ we show that increased *CETP* expression is significantly associated with lower HDL levels, and the direction of effect is the same for EN, RF, and SVR models. Thus, we computationally corroborate the biological importance of *CETP* gene in HDL-associated diseases. In many studies, the *CETP* gene has been experimentally associated with HDL levels in humans, and it currently stands as a potential drug target for the treatment of atherosclerosis.^54–58^ Thus, our analysis in a relatively small TWAS (n = 3,856) identified a known drug target that has been studied extensively in the context of preventing cardiovascular disease.

Nonetheless, there are some limitations to the practical application of the non-linear ML models like RF in comparison to linear models like EN. One of the major flaws of ensemble tree regressions such as RF is that they cannot extrapolate to data points (or ranges) they have not seen, thus restricting predictive performance of each RF model to the boundaries of the training dataset. Unlike RF, linear models such as EN and SVR with linear kernel can generate prediction values for data points beyond the boundaries of the training data because they can extrapolate well. Additionally, EN models typically expose the predictors and their corresponding effect sizes such that they are easily accessible and extractable, while RF models do not. Access and utilization of these predictors and effect sizes can make application on test datasets much easier and relatively faster. Another practical consideration is the ability of the prediction models to utilize GWAS summary statistics as input data instead of the actual genotype dataset. This is important because of the data-sharing limitations often associated with human genetic information. EN as implemented in S-PrediXcan^59^ is able to predict gene expression with only the GWAS summary statistics, while the applicability of non-linear models like RF in TWASs is limited to only GWASs with genotype and phenotype data available. As such, EN has more practical advantage than RF for genes that both algorithms can predict.

We also note that improvements in expression prediction performance beyond EN have recently been demonstrated by integrating adaptive shrinkage methods like MASHR, which improves effect size estimates across multiple experiments.^60^ Applying MASHR worked well in the context of using GTEx Project data to build gene expression prediction models because of similar eQTL effect sizes across the 54 tissues of GTEx.^61^ There might be a role for a MASHR-like framework to build cross-population models in either the same or multiple tissues, and this is a promising avenue for future research when more diverse population transcriptome data are available.

In conclusion, although linear modeling of SNPs and gene expression is generally good at imputing expression for new data, linear models may fail to accurately predict expression for some genes. Interestingly, our study shows the imputation performances for some genes are comparatively better with non-linear ML models like RF (Figure 4) than linear models like EN, especially between diverse populations. Therefore, by increasing ancestry diversity and sample sizes of study populations, optimizing prediction performance on these genes with RF modeling may be warranted. While incorporating RF models into the existing PrediXcan tool has practical limitations, doing so may be justified when genotypes are available to increase the probability of uncovering new gene-trait associations in downstream transcriptome-phenotype analyses.

## Supplementary Material

1

2

3

4

5

## Figures and Tables

**Table 1.** Mean cross-validated gene expression prediction performance of machine learning models in MESA populations

Population	EN	RF	SVR	KNN
AFA	0.0528	0.0041	0.0120	−0.0086
HIS	0.0479	0.0156	0.0289	0.0001
CAU	0.0596	0.0283	0.0437	0.0094
ALL	0.0733	0.0409	0.0476	0.0103

Elastic net (EN) had higher mean performance than each of the other machine learning models across the MESA populations (all paired t test p values < 4 × 10^−19^). AFA, MESA African American; CAU, MESA European American; HIS, MESA Hispanic American; ALL, all MESA; RF, random forest; SVR, support vector regression; KNN, K nearest neighbor.

**Table 2.** Number of genes with expression prediction models for each method after filtering by cross-validated R^2^ in the ALL cohort

Method	R^2^ > −0.1	R^2^ > −0.01	R^2^ > 0	R^2^ > 0.01	R^2^ > 0.05	R^2^ > 0.1	R^2^ > 0.5
EN	9,622	9,621	6,823	5,729	3,176	2,108	222
RF	9,544	4,924	4,158	3,651	2,449	1,687	194
SVR	9,622	8,929	5,355	3,772	2,185	1,454	141
KNN	9,263	4,193	3,206	2,601	1,422	839	28

Total gene models before filtering; EN = 9,622, RF = 9,623, SVR = 9,623, KNN = 9,623. EN, elastic net; RF, random forest; SVR, support vector regression; KNN, K nearest neighbor.

**Table 3.** Mean prediction performance of MESA-trained models in METS

Model	AFA	HIS	CAU	AFHI	ALL	Number of genes
EN	0.1123	0.0859	0.0674	0.1211	0.1185	2,097
RF	0.1217	0.1163	0.0931	0.1272	0.1265	1,574
SVR	0.1015	0.1005	0.0857	0.1144	0.1142	1,415
KNN	0.0854	0.0784	0.0684	0.0897	0.0899	1,069

We focused on the genes predicted in all 5 of the training populations for each prediction method. EN, elastic net; RF, random forest; SVR, support vector regression; KNN, K nearest neighbor; AFA, MESA African American; HIS, MESA Hispanic American; CAU, MESA European American; AFHI, MESA African American and Hispanic American; ALL, all MESA.

**Table 4.** Mean prediction performance of genes predicted in METS by all 4 of the prediction algorithms for each training population

Population	EN	RF	SVR	KNN	Number of genes
AFA	0.1210	0.1150 (p = 3.0 × 10^−3^)	0.0959 (p = 4.5 × 10^−23^)	0.0723 (p = 1.9 × 10^−47^)	1,640
HIS	0.0880	0.1066 (p = 5.3 × 10^−11^)	0.0896 (p = 5.9 × 10^−1^)	0.0648 (p = 1.4 × 10^−12^)	1,809
CAU	0.0620	0.0770 (p = 1.3 × 10^−7^)	0.0699 (p = 4.0 × 10^−3^)	0.0475 (p = 5.4 × 10^−6^)	2,091
AFHI	0.1111	0.1068 (p = 1.1 × 10^−2^)	0.0944 (p = 8.8 × 10^−16^)	0.0695 (p = 9.6 × 10^−50^)	2,290
ALL	0.1074	0.1046 (p = 1.2 × 10^−1^)	0.0944 (p = 1.3 × 10^−11^)	0.0659 (p = 9.0 × 10^−49^)	2,315

For each training population, we took only intersection genes predicted by EN, RF, SVR, and KNN. Focusing on these intersects for each training population, we calculated the mean prediction performance (ρ). The paired t test p value between EN and each other model is shown in parentheses. EN, elastic net; RF, random forest; SVR, support vector regression; KNN, K nearest neighbor; AFA, MESA African American; HIS, MESA Hispanic American; CAU, MESA European American; AFHI, MESA African American and Hispanic American; ALL, all MESA.

**Table 5.** Mean prediction performance in METS of pairwise model intersecting genes

Population	EN versus RF	EN versus SVR	EN versus KNN
AFA	0.1075 versus 0.1021 (p = 2.8 × 10^−3^)	0.1072 versus 0.0857 (p = 2.2 × 10^−20^)	0.1111 versus 0.0691 (p = 6.4 × 10^−6^)
HIS	0.0793 versus 0.0960 (p = 2.1 × 10^−11^)	0.0797 versus 0.0799 (p = 0.95)	0.0846 versus 0.0616 (p = 1.8 × 10^−13^)
CAU	0.0555 versus 0.0699 (p = 1.1 × 10^−8^)	0.0535 versus 0.0588 (p = 0.024)	0.0592 versus 0.0450 (p = 3.7 × 10^−6^)
AFHI	0.0991 versus 0.0924 (p = 8.7 × 10^−6^)	0.0975 versus 0.0815 (p = 9.9 × 10^−19^)	0.1058 versus 0.0661 (p = 1.7 × 10^−49^)
ALL	0.0909 versus 0.0871 (p = 0.017)	0.0902 versus 0.0774 (p = 2.8 × 10^−14^)	0.1041 versus 0.0628 (p = 1.5 × 10^−50^)

We performed paired t tests between the prediction performance of EN and each of the other machine learning models with each training population. The t test p values are shown in parentheses. EN, elastic net; RF, random forest; SVR, support vector regression; KNN, K nearest neighbor; AFA, MESA African American; HIS, MESA Hispanic American; CAU, MESA European American; AFHI, MESA African American and Hispanic American; ALL, all MESA.

**Table 6.** Number of ALL-trained predicted genes in METS in algorithm pairs

Genes	EN versus RF		EN versus SVR		EN versus KNN	
						
Overlap	1,198		1,141		676	
Unique	778	309	835	309	1,300	233

We only counted genes where the algorithms have significant performance (ρ > 0.1). EN, elastic net; RF, random forest; SVR, support vector regression; KNN, K nearest neighbor.
