# Supplementary material for: Transcriptome prediction performance across machine learning models and diverse ancestries
Source: HGG Adv. 2021 Jan 5;2(2):100019. doi: 10.1016/j.xhgg.2020.100019 (PMC8087249; doi:10.1016/j.xhgg.2020.100019)
Supplement: Document S1. Supplemental Figures S1–S10 and Tables S4–S6 [file mmc1.pdf]

XHGG, Volume *2*

Supplemental Information

Transcriptome prediction performance

across machine learning models and diverse ancestries

Paul C. Okoro, Ryan Schubert, Xiuqing Guo, W. Craig Johnson, Jerome I. Rotter, Ina Hoeschele, Yongmei Liu, Hae Kyung Im, Amy Luke, Lara R. Dugas, and Heather E. Wheeler

Supplemental Figures and Tables

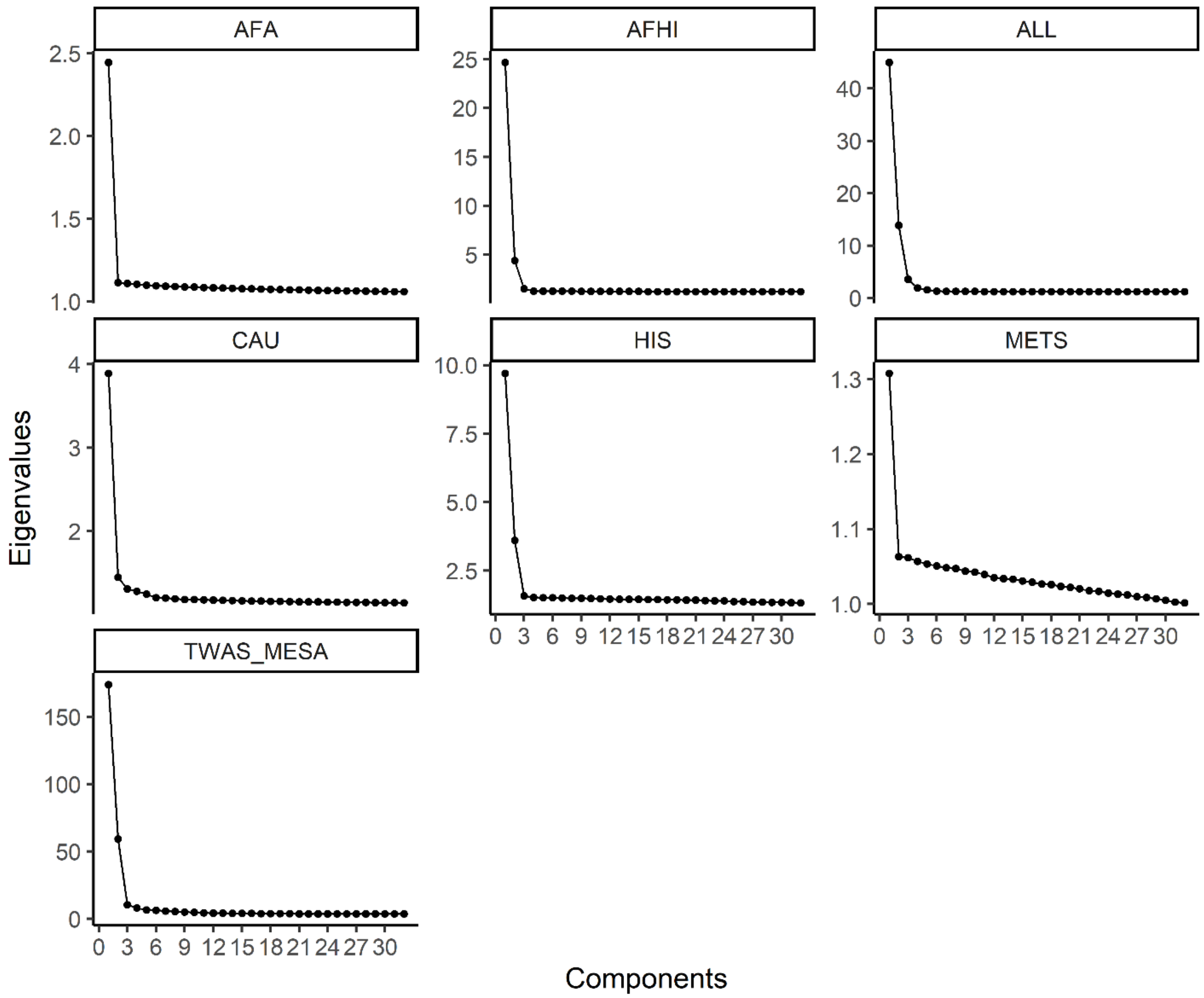

Figure S1. **Scree Plots from Genotype Principal Component Analysis of the Study Populations.** Transcriptome model training populations included AFA (MESA African American), AFHI (MESA African American and Hispanic American), ALL (all MESA), CAU (MESA European American), and HIS (MESA Hispanic American). METS (Ghanaians and African Americans) was the transcriptome test population. TWAS_MESA is the larger MESA population that was used in the TWAS analysis.

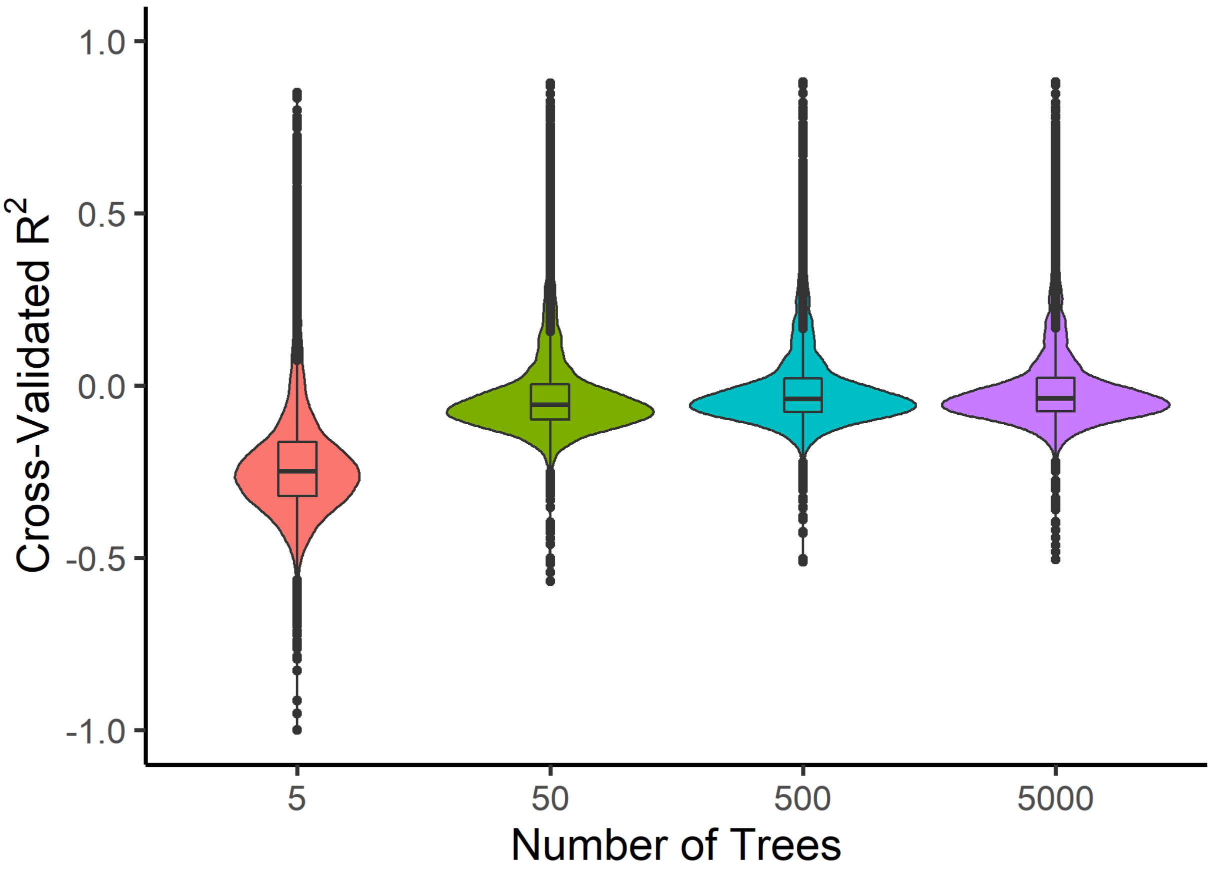

Figure S2. **Random Forest Trees Performance.** We compared the distribution of the CV R^2 of all genes at different random forest number of trees. This informed the range of trees we used in the random forest model building hyperparameter tuning. In this plot, gene models with CV R^2 < -1 were filtered out.

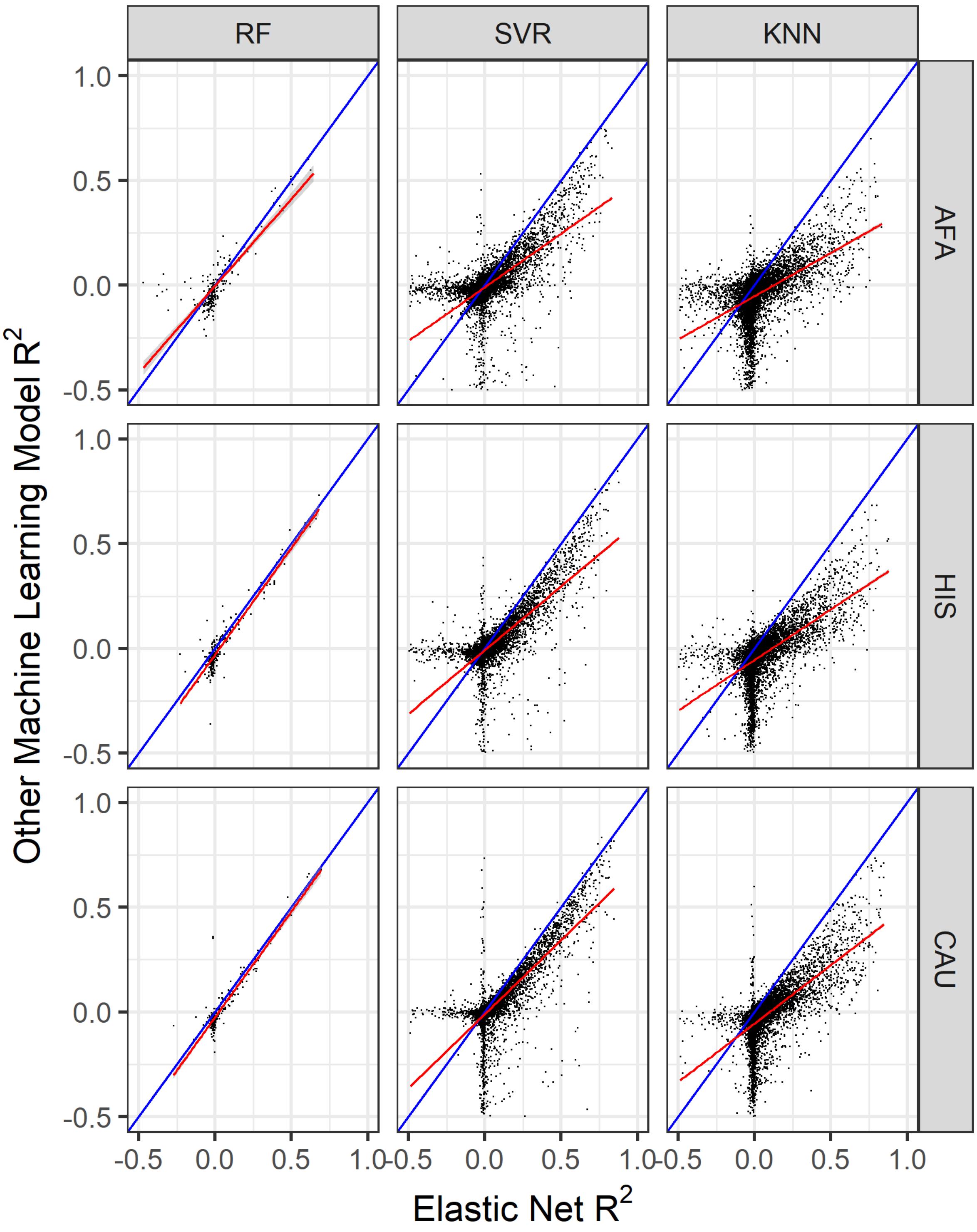

Figure S3. **Comparison of the Hyperopt Standardized Cross-Validated Gene Expression Prediction Performance in the MESA Cohort**. Machine learning (ML) models prediction R^2 compared to elastic net across MESA subpopulations. The linear regression fit is shown by the red line and the identity line (slope=1) is blue in each plot. Across the MESA subpopulation, for EN vs KNN, and EN vs SVR, we built models for all protein coding genes in chromosomes 1 to 22, while for EN vs RF, we focused only on

chromosome 22. The number of overlapping genes are as follows; AFA cohort, EN vs RF= 240, EN vs SVR= 9167, EN vs KNN = 9504; HIS cohort, EN vs RF= 243, EN vs SVR= 9044, EN vs KNN =9452; CAU cohort, EN vs RF= 242, EN vs SVR= 9094, EN vs KNN = 9483.

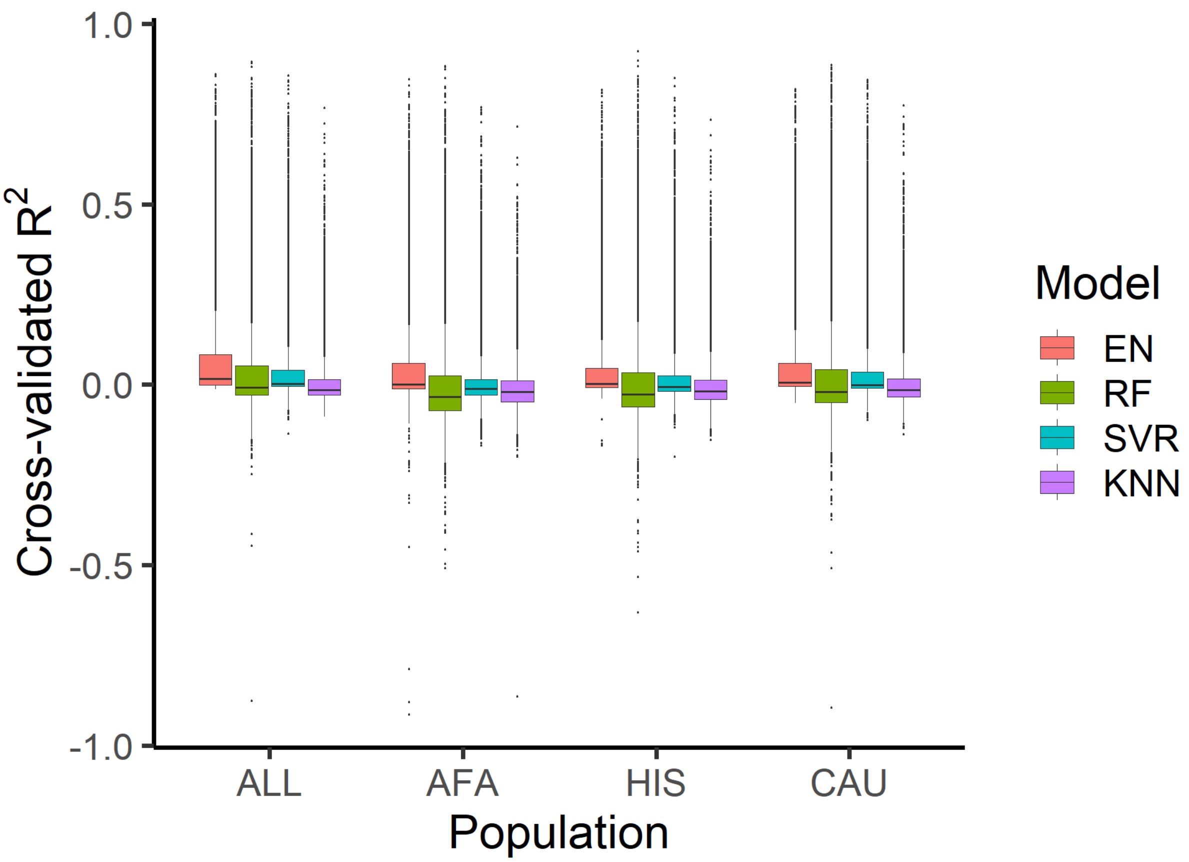

Figure S4. **Distribution of the Cross-Validated Gene Expression Prediction Performance in the MESA Cohort**. The distribution of gene models with CV $R^2 > -1$ in the ALL (EN=9622, RF=9623, SVR=9623, KNN=9623), AFA (EN=9609, RF=9622, SVR=9623, KNN=9623), HIS (EN=9621, RF=9501, SVR=9501, KNN=9501), and CAU (EN=9621, RF=9501, SVR=9501, KNN=9501) cohorts. Abbreviations are Elastic Net (EN), Random Forest (RF), Support Vector Regression (SVR), K Nearest Neighbor (KNN).

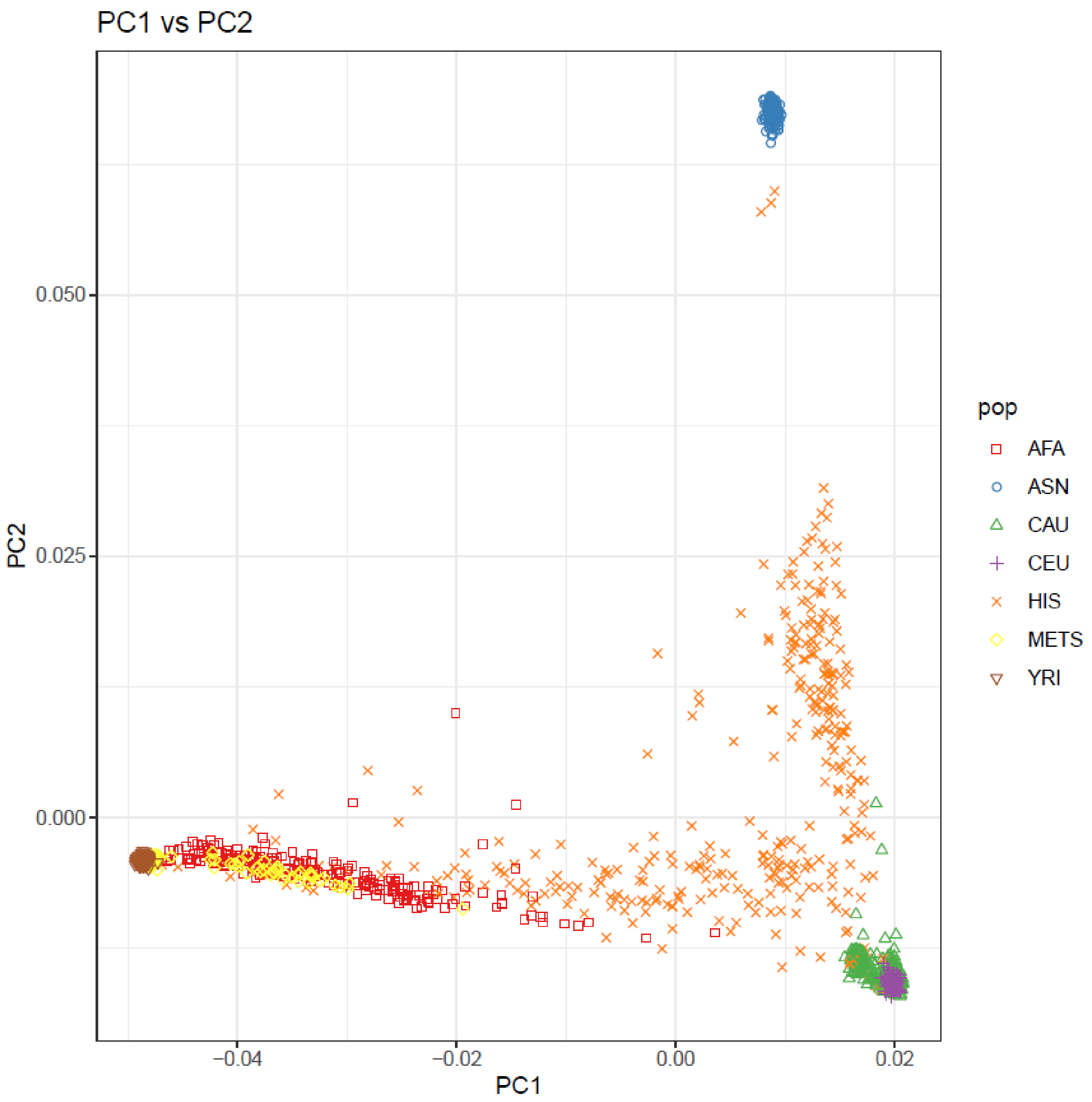

Figure S5. **Principal Component Analysis of METS.** The genotypic principal component plot of the METS (Modeling the Epidemiological Transition Study) and MESA (Multi-ethnic Study of Atherosclerosis) populations analyzed with HapMap populations. The abbreviations are MESA African Americans (AFA), East Asians from Beijing, China and Tokyo, Japan (ASN), MESA European Americans (CAU), European ancestry from Utah (CEU), MESA Hispanic Americans (HIS), Yoruba from Ibadan, Nigeria (YRI).

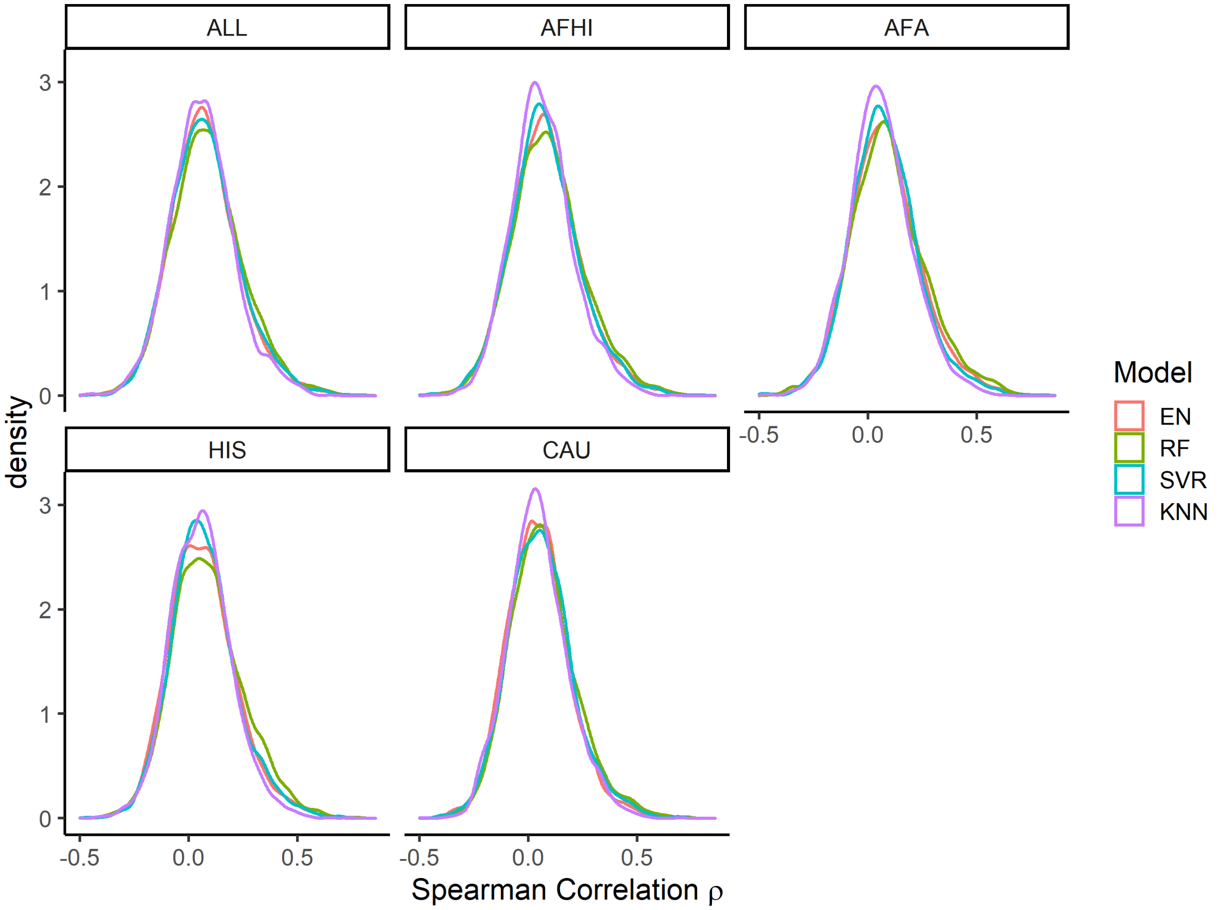

Figure S6. **Distribution of Prediction Performance in METS from Models Trained in MESA cohort.** Distributions of prediction performance (Spearman's ρ) for genes with ρ > -0.5 in each algorithm. Note, EN and RF models have similar distributions and tend to shift towards the right compared to SVR and KNN.

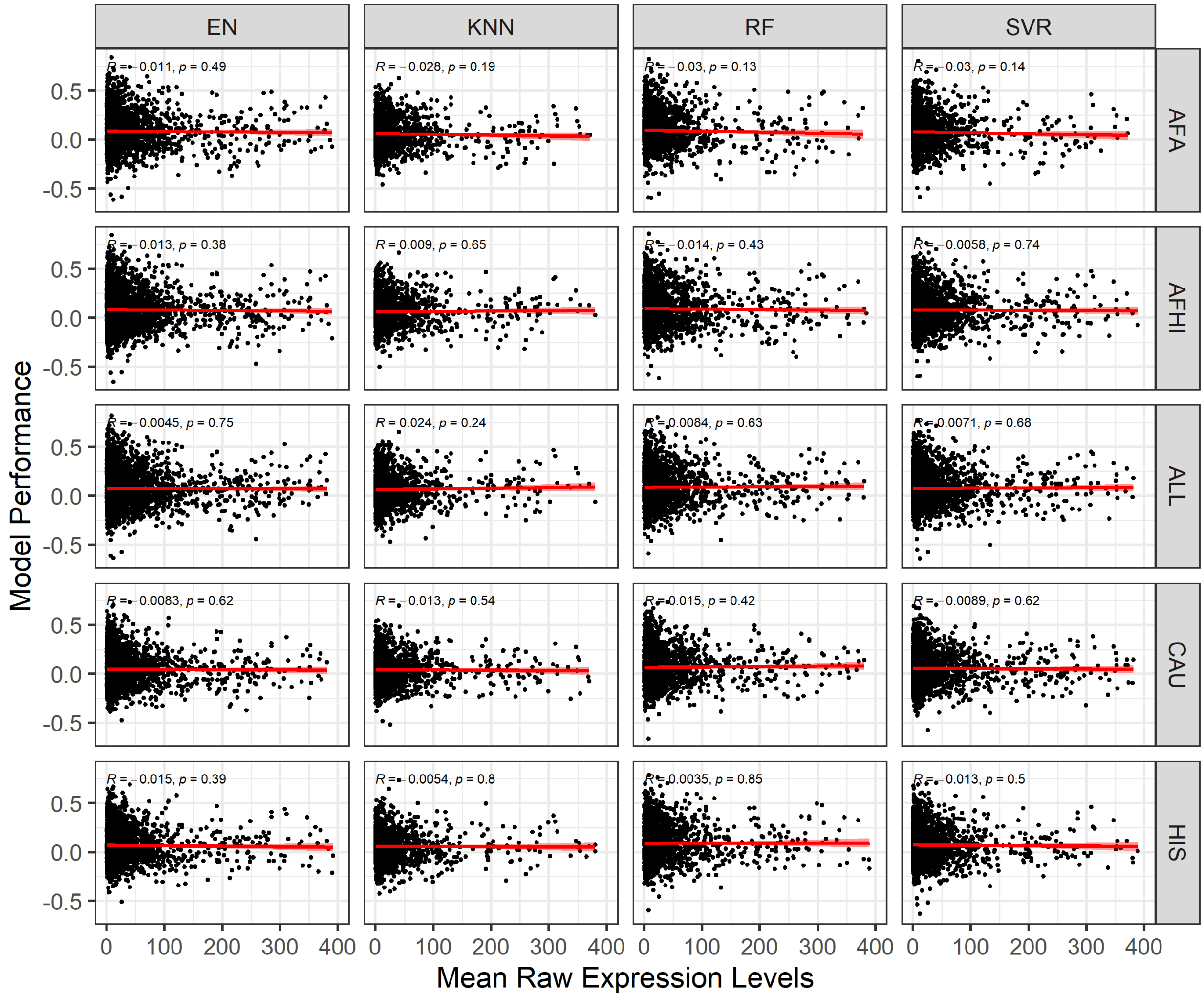

Figure S7. **Comparison of Model Performance in METS with Mean Raw Expression Levels.** Model performance ρ (Spearman correlation between predicted and observed gene expression in METS) for each gene in each machine learning (ML) model vs. the mean raw expression levels in transcript per million (TPM) is shown. The linear regression fit is shown by the red line in each plot.

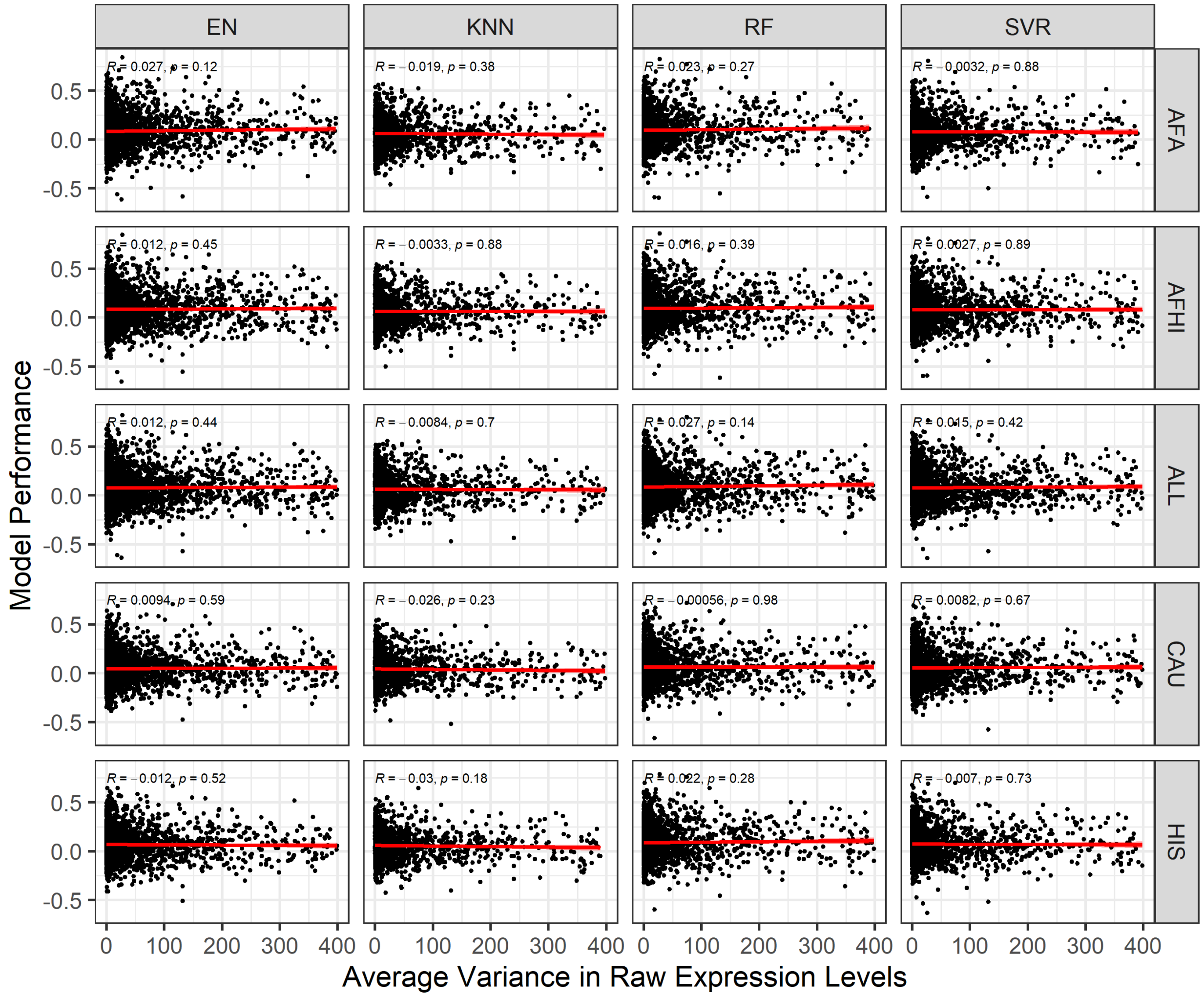

Figure S8. **Comparison of Model Performance in METS with Average Variance in Raw Expression Levels.** Model performance ρ (Spearman correlation between predicted and observed gene expression in METS) for each gene in each machine learning (ML) model vs. the average variance in the raw expression levels is shown. The linear regression fit is shown by the red line in each plot.

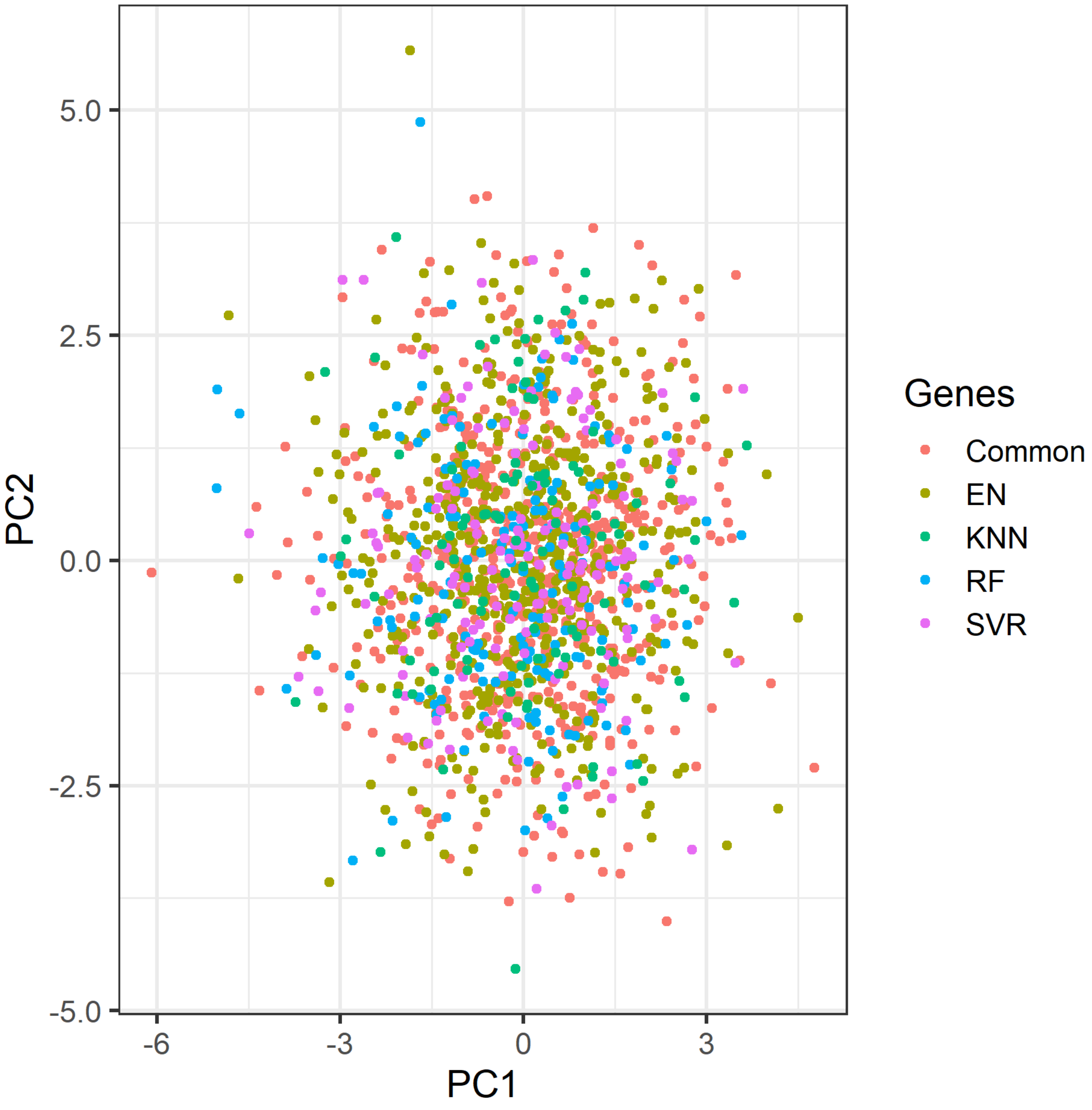

Figure S9. **PCA Cluster Analysis of the Normalized Gene Expression Levels in METS.** Principal Component Analysis (PCA) was applied to the expression levels of all the genes in the models. Each dot in the plot represents a gene, and each gene is colored based on the algorithm that captured it. Common means that a gene was captured by all the algorithms in gene expression prediction in the METS cohort.

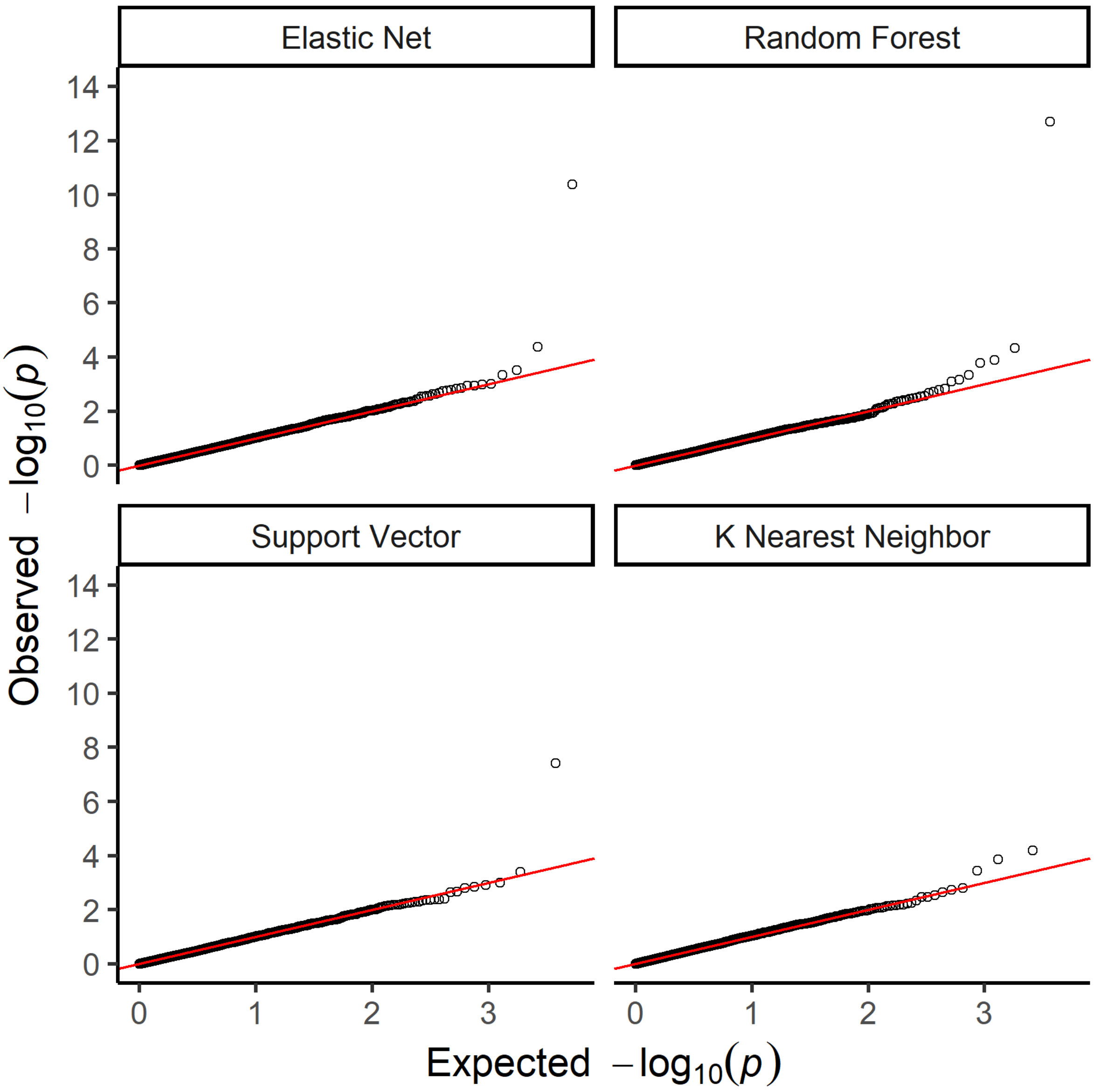

Figure S10. **Q-Q Plot of Association Tests P-Values.** Q-Q plot of the P-values from the TWAS between HDL (high density lipoprotein) values and predicted gene expression. Using models trained in MESA ALL cohort, we predicted gene expression in MESA (n=3856) genotype data comprising of individuals not used in the model training and that equally has HDL phenotype data and then carried out TWAS. The red line in each plot show the null expected distribution of the P-values.

Table S1. **Optimum Random Forests number of trees for each gene across training populations**. (txt)

Table S2. **Optimum *K* Nearest Neighbor hyperparameter combinations for each gene across training populations**. (txt)

Table S3. **Optimum Support Vector hyperparameter combinations for each gene across training populations**. (txt)

Table S4. **Number of genes with expression prediction models for each method after filtering by cross-validated R^2 in the AFA cohort**. Total gene models before filtering; EN=9623, RF=9623, SVR=9623, KNN=9623. Abbreviations are Elastic Net (EN), Random Forest (RF), Support Vector Regression (SVR), K Nearest Neighbor (KNN).

**Method**	**R^2 > -0.1**	**R^2 > -0.01**	**R^2 > 0**	**R^2 > 0.01**	**R^2 > 0.05**	**R^2 > 0.1**	**R^2 > 0.5**
EN	9589	6641	4860	4051	2601	1814	181
RF	8538	3608	3165	2841	1970	1398	157
SVR	9574	4492	3258	2648	1462	917	52
KNN	9361	3864	3093	2473	1163	581	10

Table S5. **Number of genes with expression prediction models for each method after filtering by cross-validated by R^2 in the HIS cohort**. Total gene models before filtering EN=9621, RF=9501, SVR=9501, KNN=9501. Abbreviations are Elastic Net (EN), Random Forest (RF), Support Vector Regression (SVR), K Nearest Neighbor (KNN).

**Method**	**R^2 > -0.1**	**R^2 > -0.01**	**R^2 > 0**	**R^2 > 0.01**	**R^2 > 0.05**	**R^2 > 0.1**	**R^2 > 0.5**
EN	9618	8009	5038	3959	2288	1532	147
RF	8858	3701	3295	2976	2101	1530	187
SVR	9497	5630	3841	3056	1784	1153	95
KNN	9460	3914	3135	2529	1317	716	17

Table S6. **Number of genes with expression prediction models for each method after filtering by cross-validated R^2 in the CAU cohort**. Total gene models before filtering EN=9621, RF=9501, SVR=9501, KNN=9501. Abbreviations are Elastic Net (EN), Random Forest (RF), Support Vector Regression (SVR), K Nearest Neighbor (KNN).

**Method**	**R^2 > -0.1**	**R^2 > -0.01**	**R^2 > 0**	**R^2 > 0.01**	**R^2 > 0.05**	**R^2 > 0.1**	**R^2 > 0.5**
EN	9621	9405	5758	4314	2619	1753	221
RF	9210	4025	3527	3108	2214	1577	241
SVR	9501	7084	4402	3387	2059	1396	178
KNN	9496	4089	3202	2606	1481	878	38
